# Designer diffusion media microstructures enhance polymer electrolyte fuel cell performance

**DOI:** 10.1039/d5ee03633j

**Published:** 2025-10-14

**Authors:** Rens J. Horst, Ralph van der Linde, Rémy R. Jacquemond, Baichen Liu, Antoni Forner-Cuenca

**Affiliations:** a Electrochemical Materials and Systems, Department of Chemical Engineering and Chemistry, Eindhoven University of Technology PO Box 513 5600 MB Eindhoven Netherlands a.forner.cuenca@tue.nl r.j.horst@tue.nl ralphvanderlinde92@gmail.com r.r.jacquemond@tue.nl b.liu@tue.nl

## Abstract

Gas diffusion media are essential components in polymer electrolyte membrane fuel cells and a broad range of electrochemical technologies, enabling efficient mass transport of gas and liquid, electronic and thermal conductivity, and structural integrity under compression. Conventional diffusion media, typically made from carbon fiber substrates with microporous layers, have been extensively post-treated to enhance performance; however, these approaches offer limited control over three-dimensional microstructure, particularly for advanced architectures with bimodal or gradient porosity – which can facilitate multiphase gas and liquid mass transport – and often rely on complex, multi-step processes. These limitations underscore the need for scalable, cost-effective fabrication methods capable of producing much broader geometrical features. Here, we introduce a scalable, bottom-up fabrication method based on non-solvent induced phase separation (NIPS) to produce carbon-based diffusion media with finely tunable microstructures. By systematically varying processing parameters, we generate thin, mechanically robust diffusion media with tailored in-plane and through-plane porosity, including isoporous and bimodal structures. Using microscopy, porosimetry, and electrochemical diagnostics, we correlate microstructural features with single-cell fuel cell performance, revealing their impact on water management and gas transport. We further demonstrate post-treatment strategies to enhance mass transport properties and benchmark the cost and scalability of NIPS fabrication against conventional carbon fiber-based diffusion media *via* techno-economic analysis. Our findings highlight the potential of NIPS as a versatile and industrially relevant pathway for next-generation diffusion media, offering new design freedoms to optimize fuel cell performance and reduce system-level costs.

## Abstract 2

We present a scalable method to manufacture gas diffusion media with tailored microstructures, enhancing polymer electrolyte fuel cell performance and offering a path beyond the traditional carbon fiber paradigm in electrochemical technologies.

## Graphical abstract

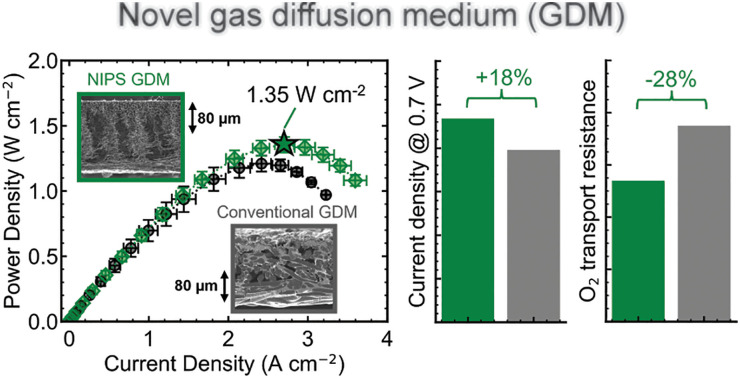

**Broader context**

The transition to a clean, low-emission energy system is one of the most pressing global challenges in the fight against climate change. Polymer electrolyte membrane fuel cells are a key technology in this transition, offering efficient, zero-emission power for applications like heavy-duty vehicles and decentralized electricity generation. However, their broader use is still limited by high system costs and performance bottlenecks. Gas diffusion media (GDM) are critical components in fuel cells, governing mass, heat, and electron transport. However, current designs are often suboptimal, relying on complex, multi-layered materials that offer limited morphological control. Our research addresses this challenge by developing a new, scalable, and more cost-effective way to engineer the internal structure of GDMs, using a process that allows to produce preferential pathways for reactant and product transport. These novel materials improve fuel cell efficiency, boosting power output by up to 16%. By enabling higher power from smaller fuel cell stacks, these improved GDMs can help lower the amount of costly materials like platinum and membrane needed - ultimately reducing the cost of clean energy systems. Our work demonstrates how advanced material design can support more affordable and widespread adoption of hydrogen-powered technologies in a carbon-constrained energy economy.

## 1. Introduction

Currently, heavy-duty transportation accounts for just 13% of the vehicle fleet in the EU, yet it is responsible for 28% of the annual transport emissions.^1^ This disproportionate contribution makes heavy-duty transport an impactful and urgent target for decarbonization efforts. Contrary to battery-electric drive trains, hydrogen-based proton exchange membrane fuel cell (PEMFC) systems benefit from independent scalability of power and energy storage capacity as well as short refueling times potentially making them more suitable for use in heavy-duty vehicles in specific use cases.^1^ Over the last two decades, PEMFC development has, however, been focused on low-duty transportation where other economic criteria dominate, mostly to lower the upfront investment costs for customers. For heavy-duty transportation, fuel efficiency and system lifetime (>30 000 h) determine the total cost of ownership and motivate a different optimization strategy.^2^ Operation at higher cell voltages while retaining identical stack power density - meaning more efficient operation - for longer time is more important than reducing Pt loading, for example. High power density at elevated cell voltages (>0.7 V) is non-trivial and requires optimization of PEMFC materials. Critical components include proton conductors,^3^ catalysts^4^ and diffusion media.^5^ There is a strong focus on the former two because they are cost-intensive, but enhancing performance using improved diffusion media can increase overall power density and thus decrease the use of other cost-intensive components. While many studies focus on improving the mass transport properties of catalyst layers to reduce the local oxygen mass transport resistance at the triple phase boundary,^6^ Kongkanand *et al.* simulated the losses in a PEMFC operating at high current density (1.75 A cm^−2^) and showed that the gas diffusion medium (GDM) still accounts for more than 25% of the mass transport voltage losses.^7^

**Fig. 1:**
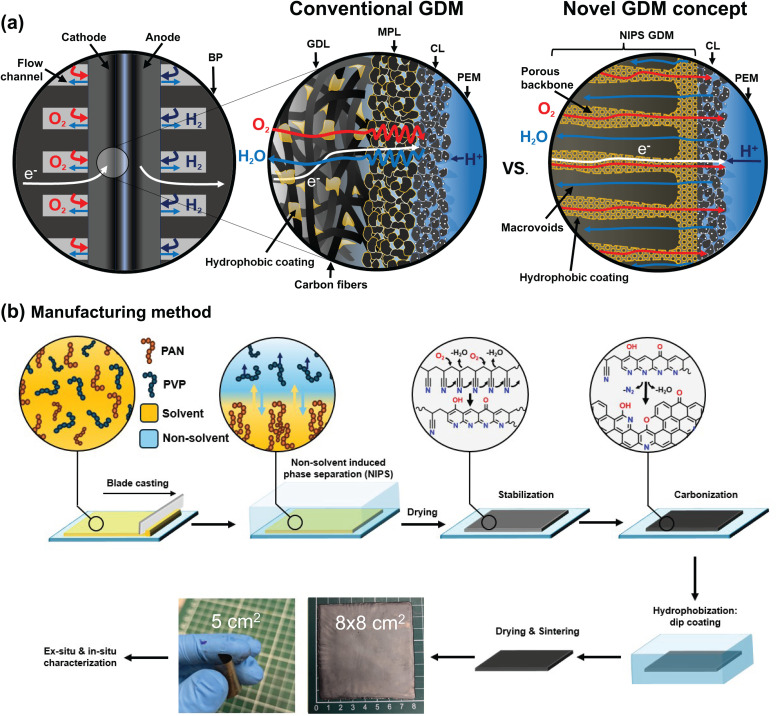
Conventional *vs.* novel GDM concept and the proposed manufacturing route (a) schematic microstructural representation of the conventional and novel GDM concept for PEMFC showing the differences in microstructure and the resulting reactant and product transport pathways. Note that the images are not to scale, as MPL porosity in reality is smaller than the NIPS GDM CL-side. (b) Overview of the manufacturing route for thin NIPS-based carbon GDMs. It starts with a solution of polyacrylonitrile (PAN) and polyvinylpyrrolidone (PVP) in dimethylformamide (DMF), which is blade cast over mold with a certain depth. Subsequently, the cast film is submerged in a non-solvent to undergo NIPS to form a porous PAN membrane. This membrane is dried, stabilized in air to crosslink the PAN and increase carbon yield and carbonized to obtain a porous carbon sheet. This material is then hydrophobized by dip coating in a PTFE dispersion, dried and the PTFE is sintered to obtain the hydrophobized NIPS GDM.

Due to the stringent requirements for two-phase mass transport, high thermal and electronic conductivity and long-lasting mechanical stability, GDMs have been specifically optimized over the years for use in fuel cells. Specific learnings from the field have also spilled over into other electrochemical technologies like water electrolysis,^8^ carbon dioxide electroreduction,^9^ and metal–air batteries.^10^ Conventional GDMs (Fig. 1a) for PEMFCs often consist of two layers:^5^ a gas diffusion layer (GDL) which interfaces with the bipolar plate (BP) and a microporous layer (MPL) which is facing the catalyst layer (CL) that sits on a proton exchange membrane (PEM). The GDL is a conductive micrometer-size carbon fiber-based porous layer (either woven, entangled or in paper form) which is hydrophobized and allows for efficient mass transport of reactants and products. The MPL consists of conductive carbon nanoparticles and a hydrophobization agent (*e.g.* polytetrafluoroethylene, PTFE) and mainly aides in the cell water management and reduces contact resistances with the CL.^11^ A GDM should sustain efficient two-phase mass transport as it must balance the supply of reactant gas with the removal of product water. At high current density operation, most of the water produced in the CL exits the cell in the vapor phase but part of it also condenses and forms liquid water pathways which compete with oxygen mass transport.^12^ Water condensation is dictated by thermal gradients inside the cell, but also depends on the pore size and hydrophobicity of the materials. In PEMFCs the pore size increases from the CL and MPL (nm range), to the GDL (μm range) and the BP (mm range). The pore size defines liquid pathways through differences in capillary pressure,^13^ so for efficient water removal, rational control of the pore size and geometry is important to increase PEMFC performance. For the gas phase, the variety in pore sizes also imply different transport modes for diffusion.^14^ Transport in the GDL is best described by multi-component molecular diffusion, whereas the MPL and CL are more subject to Knudsen diffusion and solution-diffusion in the ionomer.^15^ Many studies in the past have focused on analyzing GDM microstructures^16,17^ and their effect on overall PEMFC performance.^18–21^ While these studies have pushed the boundaries of our understanding of PEMFC operation, they are confined to the current paradigm of carbon fiber GDMs and its limited microstructural design space.

More recently, a paradigm shift can be observed to alternative materials, microstructures and cell configurations.^22^ For example, Alink *et al.* modified the microstructure of conventional GDMs by laser post-processing to introduce perforations (large pores of ∼100 μm). The approach resulted in improved oxygen diffusivity but made the material more vulnerable to flooding because the laser treatment induced hydrophilicity around the perforations.^23^ Chevalier *et al.* produced electrospun GDMs consisting out of hydrophobic nanofibers.^24^ The process of electrospinning enabled controlling fiber diameter and orientation, widening the microstructural design space. However, in a follow up paper by the same group the electrospun GDMs proved to be susceptible to degradation.^25^ To produce more stable GDMs, Choi *et al.* used a freeze casting method to produce titanium-based foam GDMs with a bimodal pore size distribution (∼15 and 40 μm pores). Their GDMs demonstrated a performance close to that of commercial GDMs and the use of titanium made the GDMs more resistant to corrosion.^26^ Analogously, Tongsh *et al.* used carbon-coated nickel metallic foams as integrated flow fields and GDMs to drastically increase stack volumetric power density by eliminating the use of separate GDMs in the stack hardware.^27^

By leveraging surface energy rather than pore size to locally modulate capillary pressure, it has been demonstrated that patterned wettability with radiation grafting can enhance through-plane water transport by establishing preferential transport pathways.^28–30^ By rational design of GDM microstructures with low tortuosity mass transport pathways, Niblett *et al.*^31^ were able to predict improvements in two-phase flow using simulations with ordered microstructures made by additive manufacturing, and concluded that bimodal pore size distributions could more efficiently segregate gas and liquid pathways. The importance of bimodality in pore size has also been highlighted in previous research and could be paramount in further improving PEMFC GDMs.^18,32^ In a later publication, Niblett *et al.*^33^ for the first time used a 3D-printed GDM in a PEMFC, but difficulties in the manufacturing route adversely affected cell performance. Similarly, Dörenkamp *et al.*^34,35^ 3D-printed GDMs with specific water extraction pathways by controlling in plane and through plane porosity. *In situ* X-Ray CT imaging indicated more efficient removal of water and better mass transport in wet conditions compared to conventional GDMs. Although this approach shows the promise of rationally designed GDMs, the complexity and relatively high cost of advanced manufacturing techniques such as micrometer-scale additive manufacturing motivates the development of economically viable production methods enabling a higher degree of microstructural control.

Inspired by these recent efforts and motivated to tackle remaining limitations, here we introduce a manufacturing method to produce novel GDM materials with engineered pathways for multiphase mass transport, while being compatible with large-scale manufacturing at competitive costs. We specifically target a microstructure with a bimodal pore size distribution as illustrated in the new GDM concept in Fig. 1a. We hypothesize that a hydrophobic carbonaceous material with large pores (>20 μm) or voids and a backbone with smaller pores (<10 μm) could provide preferential pathways for liquid and gas transport and improve fuel cell performance. According to the Young–Laplace equation, lower capillary pressure would be needed to invade larger pores, making it easier for water to enter these voids. In contrast, higher pressures are required to invade smaller hydrophobic pores, which resist water intrusion and help keep these pathways open for gas transport. We develop a facile manufacturing route based on non-solvent induced phase separation (NIPS), often employed in the production of filtration membranes.^36^ This technique was previously adapted and pioneered by our group to fabricate carbon electrodes with tunable microstructures for its use in redox flow batteries.^37^ In redox flow batteries, reactant mass transport occurs in a single liquid phase and the microstructures are optimized in terms of high active surface area and low pressure drop, making these electrodes relatively thick (>600 μm). Transferring the carbon membranes to fuel cell applications requires a shift in optimization criteria, where parameters such as electrode thickness, compressibility, and hydrophobicity become critical to facilitate multiphase mass transport. To address these limitations, we introduce – for the first time in PEMFCs – a thin (<200 μm), hydrophobic GDM with a bimodal pore size distribution, fabricated *via* the NIPS process (Fig. 1b), and benchmark its performance against a state-of-the-art commercial GDM.

In this manuscript, we first compare the novel GDM material with bimodal pore size distribution produced by controlling the NIPS synthetic parameters and a state-of-the-art conventional GDM. We use microscopy, porosimetry, spectroscopy, and fuel cell testing to understand how morphological and physicochemical properties of the new GDMs influence the cell performance. Second, to evaluate the impact of bimodal porosity on water management and fuel cell performance, we fabricate a reference GDM with a unimodal pore size distribution *via* NIPS combined with an extended vapor induced phase separation (VIPS) step and compare it to its bimodal counterpart. Third, we further tune the microstructure of NIPS GDMs using a laser post-treatment to study the influence of the NIPS interface with the CL on performance. We then compare mass transport characteristics of all different microstructures based on the *in situ* fuel cell data. Finally, we conclude with a techno-economic analysis of the NIPS GDM manufacturing process and contextualize these results with a comparison to conventional GDM manufacturing.

## 2. Materials and methods

### 2.1. Chemicals & materials

Polyacrylonitrile powder (PAN, 99.5% acrylonitrile, *M*_w_ ≈ 200 kDa and particle size of 40 μm) was purchased from Dolan GmbH. Polyvinylpyrrolidone powder (PVP, *M*_w_ ≈ 1300 kDa) and *N*,*N*-dimethylformamide (DMF, ≥99.9%) were purchased from Sigma-Aldrich and used as received. Teflon™ PTFE Disp 30 aqueous dispersion was acquired from Fuel Cell Store for hydrophobization. Regular tap water was used for the coagulation bath and washing step. Redistilled mercury (+99.9%) for porosimetry was purchased from Alfa Aesar. Casting molds were made in-house (size of 15 × 15 × 0.04 cm^3^) out of polypropylene which was weighted down with stainless steel bolts to prevent floating in the coagulation bath. A polypropylene box (30 × 40 × 20 cm^3^) was used as coagulation and washing bath. Graphite plate material (22 × 22 × 0.5 cm^3^) was ordered from JPGraphite and 3 mm thick melamine foam was ordered from Melamine Foamtech. Graphite tape, PTFE sheets (1 mm thickness), and Kapton foil was purchased from Eriks BV. Catalyst coated membranes were received from EKPO Fuel Cell Technologies GmbH consisting out of a Gore M788.12 reinforced membrane with an anode and cathode Pt loading of 0.1 and 0.4 mg Pt cm^−2^ (Fig. S1), respectively. CMC61325 PEN foil was used as subgasket material (CMC Klebetechnik GmbH). The baseline GDM material, Freudenberg FH15C14 (150 μm thick at 1 MPa, with microporous layer), was purchased from Quintech GmbH.

### 2.2. NIPS GDM manufacturing

#### 2.2.1. Polymer solution

The manufacturing process is depicted schematically in Fig. 1b and started with the addition of 200 mL of DMF to a three-necked 29/32 round-bottom flask (500 mL). A mechanical overhead stirrer with a PTFE stirring rod was inserted into the middle opening of the flask and sealed using a BOLA PTFE stirring seal. A needle which was slowly purging Ar was inserted through a septum to avoid excessive water uptake by the DMF. The DMF was first pre-heated using an oil bath to 90 °C while stirring. A pre-weighed amount of PAN (12.05 g) and PVP (24.09 g) in a ratio of 1 : 2 was slowly poured in through the remaining opening while stirring to make a 16 wt-% polymer solution. The polymers were left to dissolve for roughly 2 hours after which the still hot, now slightly yellow polymer solution was transferred to 250 mL Duran bottle. The solution was left to cool down to room temperature and to remove any entrapped air bubbles.

#### 2.2.2. Membrane casting

For casting (Fig. S2), a portion of the polymer solution was poured onto the far end of the mold and was cast using a casting knife and device built in-house. Depending on the process, either direct submersion into the coagulation bath (NIPS) or 5 min VIPS in ambient air (RH ≈ 60–70%) was performed before NIPS. A white PAN membrane was formed during NIPS and was allowed to complete phase separation for 15 minutes before being removed from the mold. The membrane was then washed in successive steps using cold and warm water to remove the residual solvent, low-molecular-weight PAN and PVP pore former still present in the structure. The membrane was left overnight in the water of the final washing step. Subsequently, the membrane was stacked between two sheets of 3 mm foam and graphite plates and dried in a vacuum oven at 60 °C for at least 2 hours.

#### 2.2.3. Thermal treatment

After removal of the water, the membranes were subjected to thermal stabilization in air using a Nabertherm P300 muffle furnace. The membranes were sandwiched between graphite plates together with spacers. The stabilization protocol consisted of a ramp of 2 °C min^−1^ to 270 °C and a hold time of 1 hour after which the oven was allowed to cool down to room temperature. The still sandwiched, now gray membranes were then transferred to a carbonization oven (Protherm ACF 170/12) and subjected to a thermal sequence under N_2_ (2 L min^−1^): room temperature to 850 °C (ramp 5 °C min^−1^), holding for 40 min, 850 °C to 1050 °C (ramp 5 °C min^−1^), hold 40 min followed by natural cooling to room temperature.

#### 2.2.4. Post-treatment and hydrophobization

The now carbonized diffusion media were then cut to 5 cm^2^ squares and either used as is for hydrophobization or subjected to a laser treatment to selectively remove the top layer. The laser treatment consists of a raster scan using a VLS3.60DT CO_2_ laser cutter from Universal Laser Systems (2.0 focusing lens, average spot size 0.13 mm). The machine settings were 10% power, 100% speed, 1000 DPI and *z*-axis 1 mm. Hydrophobization was carried out using dip coating in a 4 wt-% water-based PTFE dispersion (200 nm particle size, Fuel Cell Store) for 1 min typically resulting in a PTFE loading of 15–25 wt-% depending on the sample microstructure. Following dip coating, the samples were dried at room temperature using vacuum for 1 hour and 1 hour at 100 °C under vacuum. The samples were then thermally treated at 370 °C for 5 minutes on a hot plate between two stainless steel plates to thermally decompose the dispersing agent and sinter the PTFE particles and is the final step towards making the NIPS GDM.^38^ PTFE loading was determined by comparing the dry sample weight before hydrophobization to the weight after the sintering step.

### 2.3. Physical–chemical characterization

#### 2.3.1. Scanning electron microscopy (SEM)

Electron microscopy images were acquired using a JEOL JSM IT100 SEM using an acceleration voltage of 5 kV and probe current of 30 pA. For cross-sectional imaging, NIPS GDM samples were fractured using the straight edge of plastic tweezers. The carbon fiber GDM was subjected to ion milling to produce a clean cross-section using a Hitachi IM4000II ion miller (3 h, 4 kV acceleration voltage, 1.5 kV discharge voltage, 5 s on/2 s off, 0.10 cm^3^ min^−1^).

#### 2.3.2. X-ray photoelectron spectroscopy (XPS)

The surface chemical composition was analyzed using a Thermo Scientific™ K-alpha™ X-ray photoelectron spectrometer (XPS), equipped with a monochromatic small-spot X-ray source and a 180° double-focusing hemispherical analyzer with a 128-channel detector. To obtain the spectra, an aluminum anode (Al Kα = 1486.6 eV) source operating at 72 W and a spot-size of 400 μm. Survey spectra were measured at a constant pass energy of 200 eV and high resolution spectra were measured at 50 eV. Background pressure was set at 2 × 10^−8^ mbar. During the measurements, the pressure was set at 4 × 10^−7^ mbar Ar for charge compensation.

#### 2.3.3. Apparent contact angle measurements

Water contact angles were obtained using a Krüss DSA30S drop shape analyzer equipped with a CF04 camera and high-power monochromatic LED. Sessile drop water contact angle measurements on the GDM materials were performed for both the BP- and CL-facing side. The drop volume was set at 10 μL and the measurement was performed on 3 different spots. The apparent contact angles were extracted using the Krüss Advance software using a manual baseline and Young–Laplace fitting function.

#### 2.3.4. Mercury intrusion porosimetry (MIP)

The pore size distribution was measured with mercury intrusion porosimetry using an AutoPore IV 9500. Approximately 100 mg of the GDM sample was used in a penetrometer with a volume of 3.59 cm^3^. Pore diameters were approximated using a cylindrical pore shape model and an assumed carbon–mercury contact angle of 130° for both the advancing and receding case.^39^ Samples had to be cut to smaller dimensions to avoid possible ink-bottle effects, but for the NIPS samples this meant possible exclusion of the macrovoids in the measurement. The evacuation pressure and time were 20 μmHg and 5 min, respectively. The mercury filling pressure was 0.0141 MPa and the equilibration time used was 20 s.

### 2.4. Fuel cell hardware and characterization

#### 2.4.1. Hardware

*In situ* fuel cell measurements were performed on an in-house built test bench equipped with dedicated controlled evaporator mixers (W-202A, Bronkhorst Nederland) for dynamic humidity control and gas mixing capabilities on the anode (H_2_, N_2_) and cathode (N_2_, air). The cell hardware was a qcF FC25/100 V2.0 compression support frame with a cellFixture cf5/100 HT cell fixture (balticFuelCells GmbH) and a cell active area of 5 cm^2^. The flow fields were machined in-house using iso-molded graphite plates (IRD Fuel cells) with 7 channel serpentine flow field, 0.5 mm channel width and 0.8 mm depth, land width was also 0.5 mm. The line temperature was maintained at 90 °C to avoid any condensation and the cell operating pressure was controlled using back pressure regulators. The cell temperature was regulated using a Huber ministat 125. Electrochemical measurements were performed using a high current potentiostat (HCP-803, Biologic). Measurement automation was done *via* a trigger-based communication protocol established between a LabView VI and Biologic EC-lab software. More details on the fuel cell setup can be found in Fig. S3 of the SI.

#### 2.4.2. *In situ* fuel cell characterization

For testing, the as received CCMs were subgasketed using CMC61325 PEN foil cut to specific shape for the cell hardware using a Silhouette Cameo 4. Subgasketing was performed by heating two metal plates covered with Kapton foil to 155 °C and inserting the subgasket-CCM sandwich in between for 3 min. To avoid compressing the active area, a 1 mm thick compressible PTFE mask was used. The cell, preheated to 80 °C, was then assembled by placing a 5 cm^2^ FH15C14 on the anode flow field, followed by the subgasketed-CCM and either 5 cm^2^ FH15C14 or a NIPS GDM. The cell was inserted back into the setup and subjected to a potentiostatic break-in using 2000 sccm H_2_ and 5000 sccm air on anode and cathode, respectively, a *T*_cell_ = 80 °C, RH_a/c_ = 100%/100%, *P*_outlet_ = 1.5 bar_abs_ and *P*_compression_ = 1 MPa. The break-in consisted of 30 cycles of 0.6 V (60 s), 0.3 V (60 s), OCV (30 s) based on the work of Klug.^40^ All samples showed no further signs of improvement at the end of this break-in procedure and two unique MEAs were tested per sample type for all the electrochemical characterizations.

Potentiostatic polarization curves were measured using the same differential flow conditions (5000 nccm air/2000 nccm H_2_) as the break-in from 0.3 V to 0.65 V in increments of 50 mV and from 0.65 to 0.9 V in increments of 25 mV at three different relative humidities (100, 80 and 60%, symmetric). Potentials were held for 2 minutes at each point to allow for current stabilization, and polarization curves were constructed by averaging the current over the final 30 seconds of each measurement. Potentiostatic electrochemical impedance spectroscopy (PEIS) was performed at each potential to obtain the high frequency resistance (*R*_HF_) as the intercept with the real axis. PEIS was conducted from 100 kHz to 1 Hz using an amplitude of 10 mV, 8 points per decade and repeated 3 times to ensure steady state. After each polarization curve the cell was flushed with 2 nlpm N_2_ for 5 min on both anode and cathode.

After these measurements, the anode was flushed with 1 nlpm 5% H_2_ in N_2_ and 2 nlpm N_2_ on the cathode for another 5 min. Then the cathode flowrate was set to 50 sccm N_2_. To determine the roughness factor (*rf*), cyclic voltammetry was performed between 0.05 V to 1 V (50 mV s^−1^) for 5 cycles. Note that these measurements were conducted at 80 °C, due to limited flexibility in cell temperature of the setup. The charge under the hydrogen desorption peak was converted using 210 μC cm^−2^ Pt to obtain the *rf*. Next, the anode and cathode were flushed with 1000 sccm H_2_ and 1000 sccm N_2_ for 10 min respectively. To measure H_2_ cross-over and shorting current, linear sweep voltammetry was performed from 0.1 V to 0.8 V with a scan rate of 5 mV s^−1^. Directly after, PEIS was performed under blocking H_2_/N_2_ conditions to obtain the proton transport resistance of the cathode CL. The measurement was carried out at a potential of 0.2 V and a perturbation amplitude of 10 mV from 100 kHz to 0.1 Hz using 8 points per decade and repeated 3 times to ensure steady state. The Nyquist plots were fit to a transmission line model based on the work of Landesfeind *et al.*^41^ to obtain the proton transport resistance (*R*_H+_). After the measurement at 60% RH, the oxygen mass transport characteristics were assessed using the limiting current technique of Baker *et al.*^42^ Limiting currents were measured by linear sweep voltammetry from 0.3 to 0.01 V (0.5 mV s^−1^). Concentrations of 1, 2, 4 and 8% O_2_ in N_2_ were used on the cathode and H_2_ on the anode both at a flow rate of 2000 sccm to limit cell pressure drop. At each O_2_ concentration, the limiting current was measured at pressures of 150, 200, 250 and 300 kPa_a_.

Non-blocking impedance spectra were fitted using an open source distribution of relaxation times (DRT) fitting tool (pyDRTtools^43^) without the inductive parts of the dataset. The optimal regularization parameter was determined by the LC method that is present in the software package. After DRT fitting the peak positions, marked with black dots, are replotted onto the Nyquist plot as separate RC semi-circles together with their cumulative result to show the agreement between the experimental data and the fitting results and thus to assess the validity of the DRT fit.

#### 2.4.3. Techno-economic analysis

The model consists of several interconnected components. A semi-continuous roll-to-sheet process is modeled, as a fully continuous roll-to-roll process is considered technically unsuitable for the production of our material. In this way we stay as close as possible to the original lab based procedure. At the core of the model lies the thermal treatment oven, which governs the overall production volume. The oven capacity (1.5 m^3^) was determined based on commercially available equipment. Process times for the various thermal treatment steps were derived from our lab-scale batch process and inform the number of batches that can be run per oven per day (two batches), accounting for ramp-up and ramp-down times. A factory uptime of 90% was assumed.

The square meter output of GDM from the ovens determines the raw material demand, incorporating a 50% shrinkage factor in both width and length, as well as the lab-scale polymer mixture formulation. Raw material prices were sourced from our laboratory suppliers. Utility costs were roughly based on Dutch market prices: $0.20 per kWh for electricity (average of 2024 and 2025^44^) and $0.90 per m^3^ for water.^45^

We developed a thermal loss model for the ovens based on material heat capacity, process temperatures, insulation thermal conductivity and thickness, and process duration, under the assumption of a constant ambient temperature. The total thermal energy (*Q*_total_) required is was calculated as:*Q*_total_ = *Q*_cp_ + *Q*_*l*_
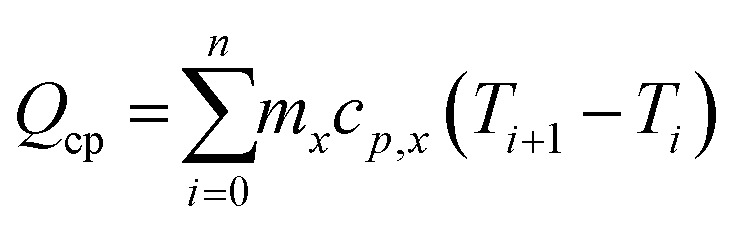

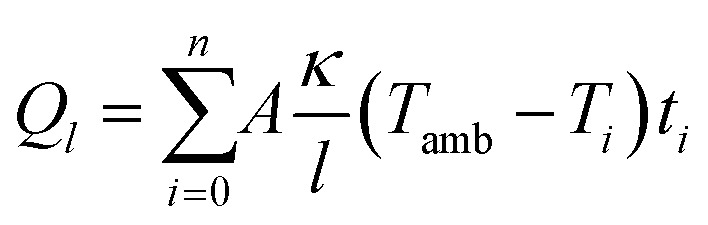
where *Q*_cp_ and *Q*_*l*_ are the energy required to heat up the oven including the materials inside and the heat loss to the environment during all the process steps, respectively. Here *m*_*x*_ and *c*_*p,x*_ denote the total mass and specific heat capacity of material *x*, and *T*_*i*_ is the temperature of the specific process step. We assume a linear temperature gradient over the insulation material and assume the outside of the insulation to be at ambient temperature (*T*_amb_), so the average temperature of the insulation material is thus assumed to be half of *T*_*i*_. In the equation for *Q*_*l*_, *A* is the insulation surface area, *κ* is the insulation thermal conductivity and *l* the insulation thickness, *t*_*i*_ is the time per process step.

The calculation for making of the polymeric solution was done based on a single helical impellor, required RPM, specific tank size and fluid specific gravity and viscosity (for details see SI Excelsheet) to estimate the required power. The heat required was determined in a similar fashion to the oven model. For the NIPS and washing step the water consumption was loosely based on the use of water in our lab per m^2^ membrane, heating of the water to 40 °C was done in a similar fashion as eqn (2). Drying of the membranes was modeled as heating of the water stuck inside the membrane plus a 1 mm water film on top to 100 °C and then evaporation of this mass of water using the latent heat of evaporation with a 70% efficiency factor for a convective air dryer.^46^ For hydrophobization we used a fixed cost per m^2^ based on the conventional GDM report,^47^ although we do model the sintering step thermal losses.

Capital expenditure (CAPEX) was calculated using a Lang factor of 4.9, reflecting the handling of both liquids and solids in the process while at the same time taking into account the high costs of thermal treatment facilities.^48^ Cost estimates for the various unit operations were derived from publicly available data on the web,^49^ unless otherwise specified in the SI Excel sheet, due to the lack of access to industrial pricing. While a comprehensive cost assessment is beyond the scope of this study, we acknowledge that this introduces uncertainty in the CAPEX calculation. For the thermal treatment ovens, we selected a specific lab-scale oven and scaled its cost by total volume using a scaling exponent of 0.65, appropriate for thermal equipment. To incorporate CAPEX into the cost per square meter of GDM, we applied a 10% return-on-investment, 2% annual inflation, and a 10-year depreciation schedule. Labor requirements were estimated at six full-time equivalent operators. Additional details, including all calculations are provided in the SI Excel sheet.

## 3. Results and discussion

### 3.1. Comparing the novel GDM to a state-of-the-art baseline

**Fig. 2:**
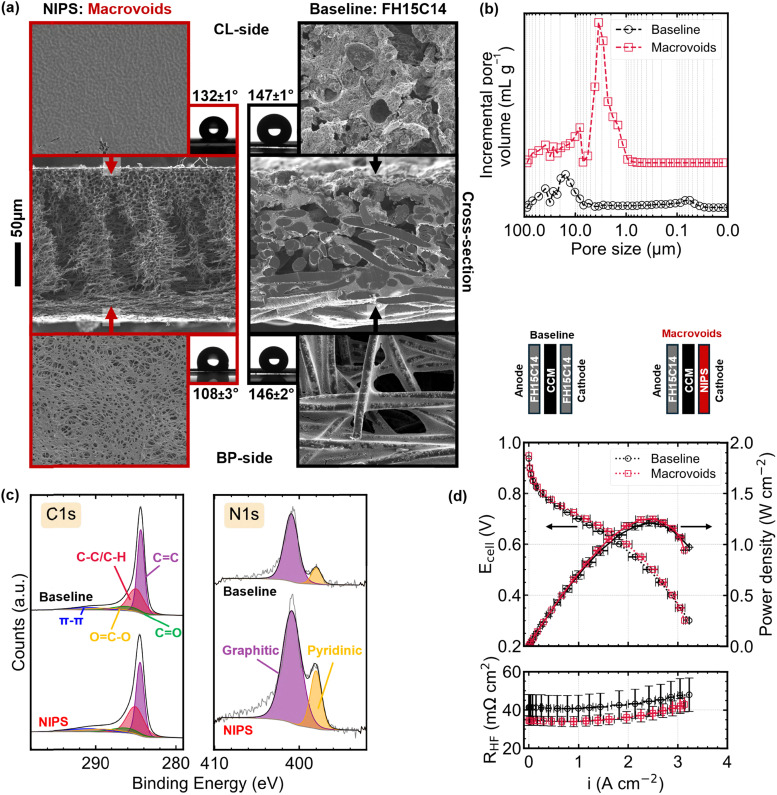
Comparison of the conventional GDM and novel NIPS-based GDM (a) SEM micrographs of the baseline FH15C14 and NIPS GDM containing macrovoids showing the CL-side, cross-section and BP-side as used in this study, all at identical magnification. Water contact angle images and values as measured by the sessile drop method are provided to give an insight in the apparent hydrophobicity. (b) Mercury intrusion porosimetry data for the baseline and the NIPS GDM showing the differences in pore size distribution. (c) Deconvoluted high-resolution XPS spectra of C 1s and N 1s to show the differences in chemical composition as a result of the lower carbonization temperature between the unhydrophobized baseline FH15 (FH15C14 without MPL and hydrophobic treatment) and the untreated NIPS material, survey spectrum in Fig. S7. (d) H_2_/air polarization curves with either FH15C14 or the NIPS macrovoids sample on the cathode side (1 MPa compression, 2/5 slpm H_2_/Air, 80 °C, 100% RH, 1.5 bar_abs_) and corresponding high frequency resistance (*R*_HF_) showing a slightly higher performance in the kinetic and ohmic regime for the NIPS macrovoids GDM (error bars are twice the standard deviation, *n* = 2).

We first set out to investigate how the microstructure of the newly developed GDM compares to that of a commercial state-of-the-art material, and how these differences translate into electrochemical performance. A comparison of the microstructural features of the novel GDM concept (NIPS Macrovoids) and a state-of-the-art baseline material (FH15C14, Freudenberg) can be seen in the electron micrographs of Fig. 2a. Cross-sectional analysis reveals that the NIPS material consists of a continuous carbon matrix featuring a bimodal pore size distribution, with large macrovoids interspersed throughout a backbone of smaller pores. The apparent bimodality is supported by mercury intrusion porosimetry (Fig. 2b) which shows intrusion in the region above 10 μm and a sharp intrusion peak centered around 3–4 μm. However, due to the nature of the microstructure, the bulk of the macrovoids will only be intruded by mercury after the bottom layer pores has been intruded, which could affect data interpretation.^39^ The thickness of the material (170 ± 5 μm) was measured at three different spots on the samples using a digital thickness gauge and we found a thickness intersample reproducibility of ±10 μm. Additionally, the thickness measured by SEM was 168 ± 2 μm. The layer which is normally oriented towards the CL – that is, the layer that was in contact with the non-solvent bath – contains a thin skin layer (∼160 nm) with occasional small pores in the range of ∼100–500 nm (Fig. S4) supported by a carbon scaffold with pores in the range of 2 to 6 μm. The side oriented towards the BP shows an open, porous structure (2–7 μm pores) which is either connected to macrovoids or the NIPS backbone. The distinct microstructures of the NIPS cross-section and the two interfaces are a result of the specific phase inversion process which has been well-studied in the field of membrane science.^37,50^

Contrary to the NIPS materials, conventional GDM's are composed of carbon fibers which are assembled into coherent structures such as papers, non-woven mats (*i.e*. *via* hydroentangling) or weaved together to form cloths.^51^Fig. 2a shows a hydroentangled carbon paper (FH15C14) which is a well-established GDM and we use as baseline in this study. The uncompressed thickness of the GDM is 171 ± 11 μm. The cross-section shows a combination of carbon fibers (diameter ≈ 10 μm) and a hydrophobizing agent combined with carbon nanoparticles (likely PTFE, Fig. S5). From the MIP data in Fig. 2b it shows that the GDM mostly features pores in the range of 10 to 100 μm. Additionally, a small intrusion peak can be observed between 50 to 100 nm which likely can be attributed to MPL present on the CL-side. The electron micrograph of the MPL shows a rough and inhomogeneous surface when compared to the relatively smooth surface of the NIPS material. Previous research has shown that the MPL roughness has a significant influence on PEMFC performance by introducing additional contact resistances and void space for liquid water accumulation.^52–54^

Fig. 2a also shows the respective external water contact angles measured on both sides of the material. The conventional GDM features a higher apparent hydrophobicity (CL-side 147 ± 2°, BP-side 146 ± 2°) compared to the NIPS materials (CL-side 132 ± 4°, BP-side 110 ± 9°) after hydrophobization according to these measurements. It should be noted however, that the PTFE application method and loading was not optimized for the specific NIPS substrate as we used a standard protocol for GDM hydrophobization that targets optimal loadings for fiber-based GDMs between 10 and 30 wt-%.^55^ PTFE loading for the NIPS GDMs was 20 ± 5 wt-%, (Fig. S6). The significant difference in surface area of the two materials complicates comparison of hydrophobicity based on PTFE loading. Adding to this, we further attribute the difference in apparent water contact angle to both the stark disparity in surface roughness of the two materials and the surface energy.^56^ As the NIPS material is carbonized at lower temperatures (1050 °C) than conventional carbon fibers (>1300 °C),^57^ the NIPS material is expected to be less graphitized. We set out to confirm this hypothesis by measuring XPS spectra of untreated NIPS and FH15 (FH15C14 without hydrophobic treatment and MPL). As shown in Fig. 2c, the FH15 contains a higher ratio of sp^2^ (C=C) to sp^3^ (C–C) carbon than the NIPS materials (roughly 2 : 1 *vs.* 3 : 2). Similarly, NIPS material also shows a stronger signal for the N 1s spectrum and a higher ratio of pyridinic to graphic nitrogen than the FH15 baseline. Overall, the NIPS GDM (91% C, 6% O, 3% N) contains a higher heteroatom content than FH15 (97% C, 1.4% O, 1.6% N) which can be attributed to its lower carbonization temperature^58^ (See Fig. S7). The increased heteroatom content increases the material surface energy and thereby potentially renders the material more susceptible to degradation processes like carbon corrosion, which is commonly observed in PEMFCs. The effect of carbon corrosion on the GDM mainly impacts cell water management as it makes the carbon surface more hydrophilic.^59,60^ Additionally, the higher heteroatom content at beginning of life also renders the material intrinsically more hydrophilic. One way to improve GDM durability would be to carbonize at higher temperatures to produce more graphitized NIPS GDMs. While it has been shown that hydrophobicity of the GDM is important for PEMFC performance,^61,62^ electronic conductivity and porosity for mass transport are often sacrificed at its expense, calling for a balanced approach when designing GDMs.^55^

To evaluate the impact of microstructure and hydrophobicity, we subjected both materials to *in situ* testing as cathode GDMs in a 5 cm^2^ active area fuel cell under 100% relative humidity and differential conditions, assessing their performance in wet operating environments. The H_2_/air polarization curve is shown in Fig. 2d and shows that the NIPS GDM shows appreciable polarization performance under humid conditions. The NIPS GDM outperforms the baseline despite the higher apparent hydrophilicity (*i*_0.7 V_ = 1.15 ± 0.04 A cm^−2^*vs.* 0.99 ± 0.06 A cm^−2^, 16% higher). In terms of peak power density (*P*_max_), the differences are smaller, but the NIPS GDM still performs slightly better (*P*_max_ = 1.25 ± 0.02 W cm^−2^*vs.* 1.21 ± 0.03 W cm^−2^, 3.3% higher). Comparison beyond peak power density makes no practical sense but shows that the baseline overtakes the NIPS GDM in the limiting current region. We hypothesize that this is due to the more closed CL-side interface which could limit oxygen diffusion.

Furthermore, Fig. 2d shows that the high frequency resistance (*R*_HF_) of the NIPS GDM is lower than that of the baseline, which is somewhat counterintuitive given that the NIPS GDM contains less graphitized carbon, as evidenced by the XPS data in Fig. 2c. We measured the *ex situ* through-plane electronic resistance (Fig. S8) and find that the continuous network of less graphitic carbon shows a higher electronic resistance (34.2 ± 0.3 mΩ cm^2^ at 1 MPa) than the discontinuous more graphitized carbon fiber matrix (16.5 ± 0.1 mΩ cm^2^). However, when testing a hybrid configuration using one NIPS GDM and one FH15C14, the resistance drops to a value (18.1 ± 0.4 mΩ cm^2^) close to that measured with two FH15C14 GDMs, suggesting that contact resistances are the dominant factor when both GDMs are of the NIPS type. The high frequency resistance (*R*_HF_) comprises several components, including contact resistances, electronic resistances, and membrane ionic conductivity. We hypothesize that the lower *R*_HF_ observed during *in situ* measurements with the NIPS GDM may stem from reduced contact resistance at the GDM–CCM interface, potentially due to its lower surface roughness or differences in water management that influence membrane hydration.

Although both materials have a similar uncompressed thickness (∼170 μm), their *in situ* performance is comparable despite their vastly different microstructures, and the lack of microporous layer in the novel NIPS GDM, which is a promising result. We poise that testing under differential flow testing conditions can partially explain these similarities. A more comprehensive comparison would ideally involve large-scale stoichiometric testing, however this is beyond the scope of this work and our testing capabilities. Overall, the macrovoid-rich GDM demonstrates strong potential in terms of performance, particularly considering the simplicity of the method with respect to commercial materials. Nevertheless, the higher oxygen mass transport limitations observed in the limiting current regime indicate that further microstructural optimization is needed. Specifically, the presence of a skin layer (*i.e.* an almost dense layer of *ca.* 100 nm challenges oxygen mass transport into the catalyst layer and constitutes an interesting feature to remove. One advantage of NIPS method is the broad microstructural design space,^37,50^ which motivated us to continue tailoring the GDM microstructure to further enhance PEMFC performance and remove the potentially inhibitive skin layer.

### 3.2. Synthetic strategy to remove the skin layer

**Fig. 3:**
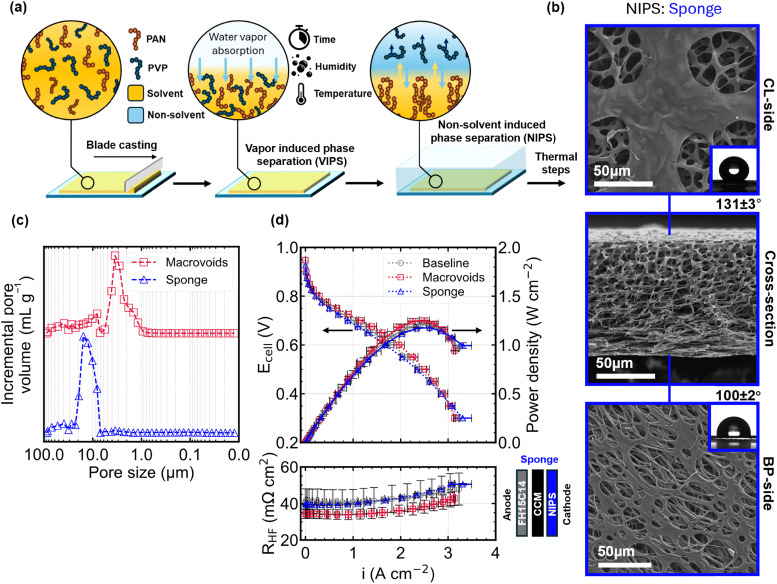
Using VIPS as a strategy to synthetically remove the top layer (a) schematic representation of the vapor induced phase separation (VIPS) process which can be used to alter the NIPS top layer morphology. (b) SEM micrographs showing the side interfaced with the CL, BP and cross-section revealing a sponge-like microstructure. Insets are apparent hydrophobic contact angles measured by sessile drop method. (c) Mercury intrusion porosimetry (MIP) data showing the differences in pore size distribution between macrovoids and sponge GDMs. A clear difference in primary pore size can be observed, with the sponge-like GDM having larger pores in general. (d) H_2_/air polarization curves (1 MPa compression, 2/5 slpm H_2_/air, 80 °C, 100% RH, 1.5 bar_abs_) and corresponding high frequency resistance (*R*_HF_) showing a slightly lower performance in the kinetic and ohmic regime, but a marginally higher performance in the mass transport limited regime.

To eliminate the skin layer at the interface facing the catalyst layer (CL), we introduced an additional processing step – vapor-induced phase separation (VIPS)^63^ – as illustrated in Fig. 3a. Prior to submerging the blade cast film into the non-solvent bath to undergo NIPS, the film was first exposed to humid air for 5 min (60% RH, 21 °C). We found that careful control of processing parameters is important to achieve reproducible microstructures and consistent material thicknesses. The VIPS process facilitates the absorption of non-solvent (water) into the top layer of the cast film, rendering it polymer-lean due to the higher mobility of solvent molecules compared to the larger polymer chains. The decrease in polymer concentration in the top layer causes a local increase in porosity upon complete phase inversion in the coagulation bath and results in a more open top layer.^64^

The resulting sponge-like microstructure of the material subjected to 5 min of VIPS is shown in Fig. 3b. The cross-sectional image reveals two notable differences: a reduced sample thickness of 102 ± 12 μm and the complete absence of macrovoids. While the initial casting thickness (400 μm) was the same as the NIPS macrovoids GDM, the 5 min VIPS process results in an uncompressed thickness 40% lower than without VIPS. This is likely a result of the VIPS time used being close to the time needed to transition from macrovoid microstructure to the isoporous sponge like structure.^65^ In the field of membrane science it is known that employing VIPS generally leads to more symmetric and isoporous membranes, while instantaneous NIPS is more likely to form asymmetric membranes with macrovoids due to spontaneous demixing.^66^ The CL-side micrograph (Fig. 3b) shows a clear openings (15–60 μm) of the skin layer and the BP-side shows a similar structure as the macrovoids sample albeit with visibly larger pore openings (7–40 μm) than the sample without VIPS (2–7 μm). Mercury intrusion data in Fig. 3c shows a broad unimodal intrusion peak for the sponge-like GDM between 22 and 7 μm centered around 16 μm. Although literature reports on the effect of VIPS on pore size vary, it is generally recognized that VIPS can promote the formation of larger pores,^67^ which is consistent with the pore size distribution obtained with MIP.

Previous studies have shown the overall positive effect of using thin GDMs,^68–70^ which is mostly attributed to the shorter dry diffusion length for reactants. However, the *in situ* performance of the sponge-like GDM in Fig. 3d shows slight underperformance compared to both the baseline and the macrovoid GDM in the kinetic and ohmic regime of the polarization curve (*i*_0.7V_ = 0.89 ± 0.03 A cm^−2^). Even though the *R*_HF_ at 0.7 V (39.4 ± 0.5 mΩ cm^2^) is similar to that of the baseline (40.7 ± 7 mΩ cm^2^), it is likely that the stark microstructural differences, and particularly the large openings on the skin-layer of the CL-side interface could introduce additional contact resistances with the CL. This is supported by the lower measured Pt roughness factor (Table 1) of the sponge GDM (107.2 ± 9.2 cm_Pt_^2^ cm_MEA_^−2^) and considerably rougher FH15C14 baseline (110 ± 4.8 cm_Pt_^2^ cm_MEA_^−2^)) compared to the macrovoids GDM sample (122 ± 4 cm_Pt_^2^ cm_MEA_^−2^) with a more even GDM-CL interface. The in-plane CL electronic conductivity combined with the lower contact area as a result of the high GDM-CL interfacial roughness could potentially influence the measured accessible Pt surface area. Another possible explanation is the effect of catalyst conditioning, where differences in GDM microstructure may lead to varying degrees of catalyst deactivation, as also suggested by Gao *et al*.^71^ It is important to note that the VIPS method not only opens the top layer but also alters the overall microstructure and thickness of the GDM. This makes it challenging to isolate and evaluate the specific impact of top-layer removal on fuel cell performance. Motivated by this, we further explored alternative strategies to remove the top layer while retaining the original macrovoid-containing morphology.

**Table 1:** Technical parameters and *in situ* fuel cell data

	Baseline	Macrovoids	Macrovoids TLR	Sponge
Thickness uncompressed^a^ (μm)	171 ± 11	171 ± 5	171 ± 5	102 ± 12
Areal density (g m^−2^)	91 ± (−)	42 ± 8	42 ± 8	27 ± 2
Porosity (−)^b^	0.74	0.89 ± 0.02	0.89 ± 0.02	0.88 ± 0.01
Through-plane permeability^c^ (μm^2^)	0.071	0.019	0.046	0.235
*R*_HF_@0.7 V (mΩ cm^2^)	40.7 ± 7.0	34.0 ± 2.5	39.5 ± 3.0	39.4 ± 0.5
*i*_0.7 V_ (A cm^−2^)	0.99 ± 0.06	1.15 ± 0.04	1.17 ± 0.04	0.89 ± 0.03
*R*_H+_ (mΩ cm^2^)^d^	74 ± 8	63 ± 6	75 ± 4	68 ± 9
*rf* (cm_Pt_^2^ cm_MEA_^−2^)^e^	110 ± 4.8	122 ± 4	121.6 ± 1.8	107.2 ± 9.2

a Measured by analyzing SEM cross-sections.

b Estimated based on the theoretical density of graphite of 2.267 g cm^−3^.

c Calculated based on capillary flow porometry results, SI Fig S7.

d Calculated using a transmission line model applied to H_2_/N_2_ EIS impedance data, SI Fig. S12.

e Extracted from *in situ* Pt cyclic voltammetry using the hydrogen desorption peak.

### 3.3. Elucidating the role of the dense top layer by post-treatment removal

**Fig. 4:**
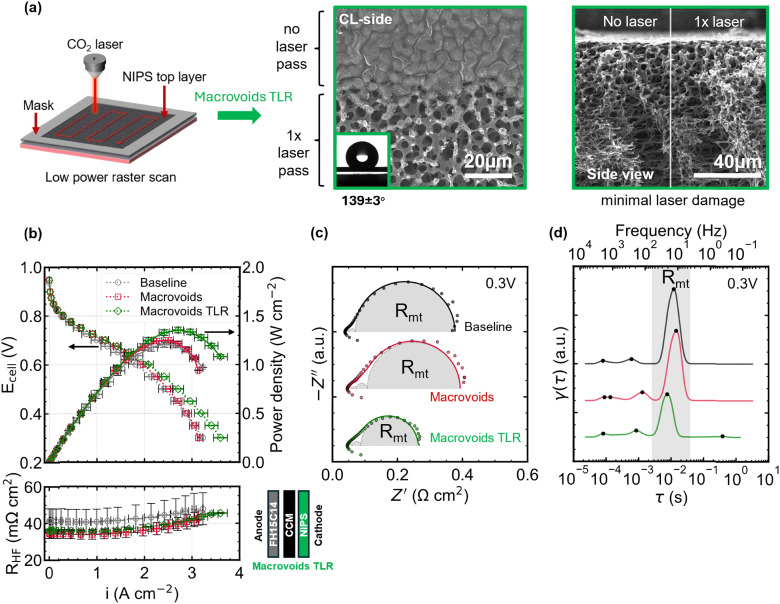
Laser ablation to remove the top layer and the effect on performance (a) schematic representation of the low power laser ablation process to selectively remove the top layer forming the macrovoids TLR (top layer removed) GDM and SEM micrographs of the top layer (CL-side) and cross-section after one laser pass showing the successful removal of the dense skin layer without significantly altering the underlaying pore structure. (b) H_2_/air polarization curves (1 MPa compression, 2/5 slpm H_2_/air, 80 °C, 100% RH, 1.5 bar_abs_) and corresponding high frequency resistance (*R*_HF_) showing a slightly higher performance in the kinetic and ohmic regime compared to the sample with an intact top layer, but a significantly higher performance in the mass transport limited regime likely due to the absence of the thin diffusive barrier. (c) Nyquist plots recorded in the mass transport limited regime (0.3 V) showing the differences in semi-circle belonging to mass transport (*R*_mt_) (d) shows the DRT result for the optimal regularization parameter. The peak related to mass transport (*R*_mt_) is indicated. All peaks, marked with black dots, are replotted onto figure (c) together with their cumulative result to show the agreement between the experimental data and the fitting results, see Fig. S10 for residuals.

To isolate the influence of the closed skin layer without substantially modifying other microstructural features, we applied a selective laser ablation post-treatment. The procedure and corresponding electron micrographs are presented in Fig. 4a. The electron micrographs show that one pass of the laser is sufficient to remove the skin layer and expose the underlying microstructure. The slight increase in apparent water contact angle from 132 ± 1° to 139 ± 3° by the removal of the skin layer is likely caused by the introduction of Cassie–Baxter states.^72^ Furthermore, the cross-sectional micrograph shows no signs of depth etching by the laser, confirming that the underlying structure is unaffected by the laser. XPS analysis (Fig. S9) showed a change in surface chemistry, with an increase in heteroatom content which could impact interfacial water management and potentially alter the material contact resistance and durability. In future work, it would be interesting to study the effects of these alterations at the GDM-CL interface on the cell water management.

We measured capillary flow porometry of the GDM materials (Fig. S11) to assess the effect of the top layer removal on the *ex situ* mass transport properties. The through-plane permeability increases significantly from 0.019 to 0.046 μm^2^ (142% increase) by removing the top layer. To evaluate the impact on *in situ* performance, we measured H_2_/air polarization curves and high frequency resistance (*R*_HF_), as shown in Fig. 4b. The results demonstrate enhanced performance of the macrovoids TLR (top layer removed) GDM compared to both the baseline and the untreated macrovoids GDM. The performance in the kinetic and ohmic regime shows a small improvement (*i*_0.7 V_ = 1.17 ± 0.04 A cm^−2)^ relative to the sample with top layer (*i*_0.7 V_ = 1.15 ± 0.04 A cm^−2^) which is further supported by the similar *rf* and *R*_HF_ values. Strikingly, the peak power density (*P*_max_ = 1.35 ± 0.03 W cm^−2^) is 8% higher than the sample with top layer, possibly related to the lower oxygen mass transport resistance caused by the removal of the skin layer as was also measured by the *ex situ* capillary flow porometry (see Table 1).

Although potentiostatic electrochemical impedance spectroscopy (PEIS) complicates the interpretation of kinetic processes – where small perturbations in potential can induce large changes in current due to the system's low impedance – galvanostatic control is generally preferred in this regime.^73^ However, in this study, PEIS is employed to probe the mass transport arc, which arises in the higher impedance region of the polarization curve, where current density becomes limited by oxygen diffusion.^74^ Judging from the Nyquist plots recorded at 0.3 V *vs.* RHE (Fig. 4c) and the corresponding relaxation times as determined by DRT analysis (Fig. 4d), the baseline and macrovoids GDM exhibit a larger *R*_mt_ arc than the macrovoids GDM without the skin layer, which further supports the hypothesis that removal of the skin layer is beneficial for mass transport. DRT analysis enables the deconvolution of Nyquist plots into their characteristic relaxation times (RC time constants), corresponding to processes occurring across different frequency ranges in EIS measurements. This allows for the isolation of the semicircle associated with mass transport limitations.^75^ It would be interesting to perform measurements on large scale stacks to confirm the positive impact of skin layer removal on mass transport and water management in technical size cells or short stacks.

### 3.4. Assessing diffusion media mass transport using limiting current measurements

**Fig. 5:**
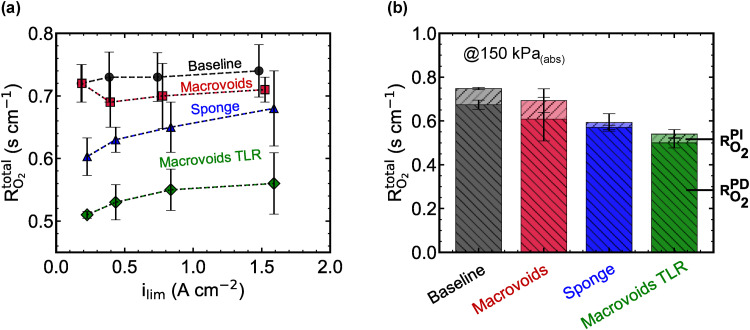
Comparing oxygen mass transport resistances between GDMs (a) total oxygen mass transport resistance 
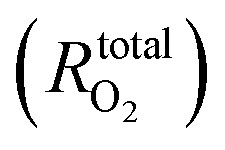
 as measured at four different O_2_ concentrations (1, 2, 4 and 8%) by the oxygen limiting current method first proposed by Baker *et al.*^42^ measured at 100% relative humidity. (b) Breakdown of of 
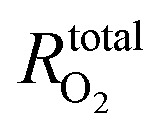
 into the pressure dependent 
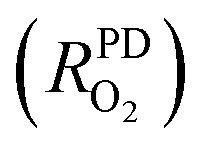
 and pressure independent 
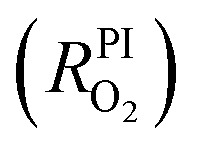
 oxygen mass transport resistance at _2_% O_2_ in N_2_ at 150 kPa_abs_ for all different GDMs.

In an effort to further elucidate the differences in mass transport characteristics we also performed oxygen limiting current experiments. Fig. 5a shows the total oxygen mass transport resistance 
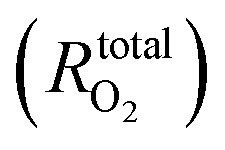
 as measured during oxygen limiting current experiments based on the method of Baker *et al.*^42^ for four different oxygen concentrations (1, 2, 4 and 8% O_2_ in N_2_). In general, the NIPS GDMs achieve a lower 
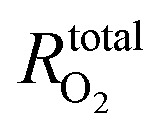
 than the baseline likely due to its completely different microstructure. The fact that 
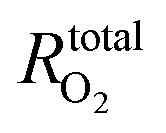
 is relatively constant over the different O_2_ concentrations shows minimal effects in terms of water management on the measurement. Although one could argue that 
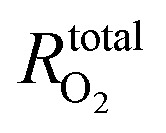
 slightly increases with rising O_2_ concentration for the sponge GDM – suggesting possible water management challenges – this remains difficult to confirm without advanced *in situ* characterization techniques such as X-ray tomography^12,76^ or neutron imaging.^77,78^ These analyses lie beyond the scope of the present study but will be the focus of future work.

In their paper, Baker *et al.* show that 
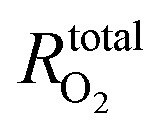
 can be deconvoluted into pressure dependent 
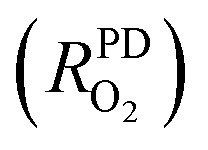
 and independent 
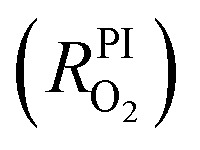
 mass transport resistance by measuring the oxygen limiting current as a function of pressure. 
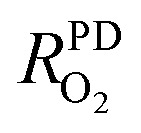
 is generally attributed to limitations in molecular diffusion, whereas 
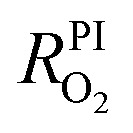
 originates from mass transport limitations attributed to Knudsen diffusion (pores < 100 nm) and solution-diffusion type mechanisms (*e.g.* diffusion through the thin ionomer film in the CL).^7^Fig. 5b shows the deconvolution of 
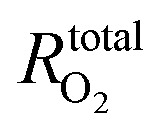
 into the pressure dependent 
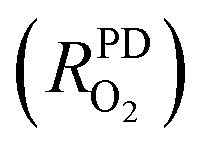
 and independent 
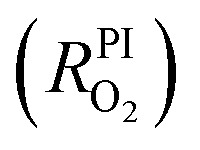
 mass transport resistance for the different GDMs we evaluated. The graph shows that the lower 
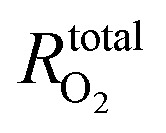
 of the NIPS GDMs can be caused by a decrease in 
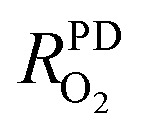
 and/or 
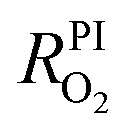
 depending on the sample type. All NIPS GDMs show a lower total dry oxygen diffusion resistance, but the macrovoids GDM only shows a decrease in 
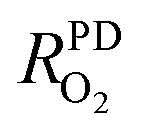
 and the difference with the baseline is likely caused by the differences in microstructure like porosity and tortuosity. It is difficult to conclude from our results whether the bimodality in pore size plays a role in this by comparing macrovoids with sponge as the latter GDM is considerably thinner than the macrovoid samples. Normalized for thickness, the sponge GDM features a 56% higher 
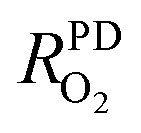
 than the macrovoids GDM, but future efforts should study changes in GDM microstructure under compression. Interestingly, the TLR sample shows a significant decrease in 
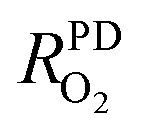
 relative to the sample with a top layer. Considering that the top layer has visible pores in the range 100 to 500 nm (Fig. S4), we find that removing the top layer has positive effect on the molecular diffusion. Aside from the drop in 
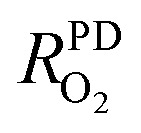
, 
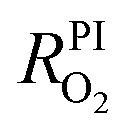
 for the TLR GDM also decreased. This indicates that the skin layer might also include pores in the Knudsen regime, because the removal of the thin skin layer would then contribute to lowering 
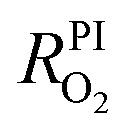
. From our data it is difficult to conclude what is the exact effect of the changes in surface chemistry caused by the laser treatment, but we expect that this more oxidized interface could play a role in the cell water management. As the skin layer can also act as a barrier for water transport or function as a potential capillary trap for water if it is not properly hydrophobized, we hypothesize that the concomitant absence of water and thus less solution-diffusion transport could cause 
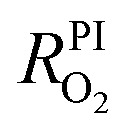
 to decrease, which opens exciting opportunities for future work on tailoring NIPS GDM microstructures at a plurality of length-scales.

### 3.5. Techno-economic analysis shows the advantages of NIPS GDMs

A key goal of this study was to develop a simpler and more cost-effective method for producing tunable GDMs, as an alternative to the complex and energy-intensive processes used in conventional GDM manufacturing. To test this hypothesis, we developed an order-of-magnitude techno-economic model based on an envisioned semi-continuous production process illustrated in Fig. 5a, which shows a schematic representation of a semi-continuous production line to fabricate hydrophobic NIPS GDMs. This production line formed the basis of the techno-economic model. More information can be found in the Methods section and the SI (including an Excel file to run the model).

**Fig. 6:**
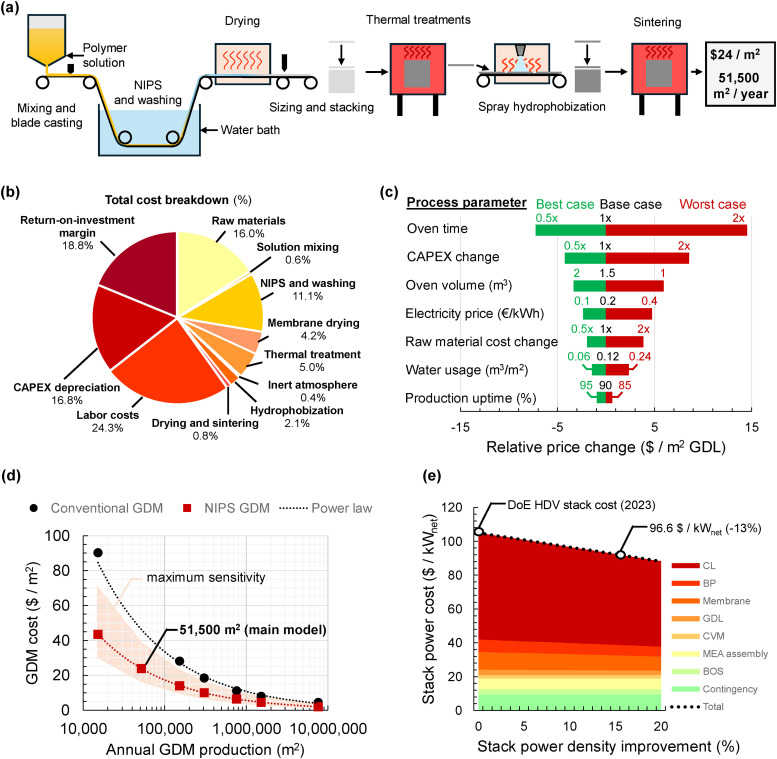
Techno-economic analysis of NIPS GDMs (a) schematic representation of the roll-to-sheet process model for NIPS GDM production resulting in a production of 51 500 m^2^ GDM per year at $24 per m^2^. (b) Pie chart showing a cost breakdown for the process model per m^2^ of GDM. Major components are the CAPEX depreciation, ROI (Return on investment) margin and various thermal treatments. (c) Sensitivity plot of different process parameters based on realistic estimates for best, base and worst case scenarios to show the potential impact on GDM cost. (d) Graph showing the effect of economy of scale on conventional GDM costs extrapolated from James at al.^47^ and power law fit which was used to extrapolate the effect of economy of scale on the costs of the novel GDM concept. The maximum sensitivity area is based on the cost change for the most influential parameter (oven time). (e) Graph showing the net stack power cost based on the 2023 DoE HDV report^79^ with an extrapolation of the cost decrease when using the novel GDM to improve stack power density at 0.7 V.

The production process begins with mixing and dissolving the materials in a heated, stirred tank to form a homogeneous polymer solution. This solution is then blade cast as a thin film onto a conveyor belt, which carries it through a non-solvent bath and drying station. The dried film is subsequently cut to size and stacked for thermal stabilization and two-step carbonization in a single oven, yielding the final carbon scaffold. Subsequently, the carbon scaffold is spray-coated with a PTFE dispersion—a deviation from our laboratory-scale dip-coating approach, as dip-coating is considered impractical at industrial scale for NIPS-derived GDMs. After coating, the substrate undergoes drying and PTFE sintering to form the final NIPS GDM. Our modeled production line yields an annual output of approximately 51 500 m^2^, with a techno-economic analysis estimating a production cost of roughly $24 m^−2^. A cost breakdown can be seen in Fig. 6b. It is important to note that capital expenditure (CAPEX) depreciation over 10 years, along with the assumed annual return on investment (ROI) of 10%, contribute significantly to the overall production costs – accounting for 16.8% and 18.8% of the total cost per square meter, respectively. The remaining costs are associated with the operational expenditures (OPEX) of the process, with labor (24.3%) and raw materials (16%) representing the largest contributions. The majority of the remaining OPEX is attributed to thermal processes, including hot water washing (11.1%), drying (4.2%), and thermal treatments (5%).

Acknowledging that our model relies on a number of approximations, we performed a sensitivity analysis of some of the main parameters governing the material costs, illustrated in the tornado plot in Fig. 6c. At the core of the model lie the carbonization oven capacity and thermal processing time, which together dictate annual production throughput. Given the batch nature of the process, thermal processing time emerges as a critical cost driver and a key target for optimization. Another important factor is CAPEX variability, stemming from uncertainty in equipment costs. Notably, CAPEX and return-on-investment together account for 35.6% of the cost per square meter. As expected for an energy-intensive process such as carbonization (∼23 kWh m^−2^ of GDM), energy prices also have a substantial influence on overall costs. At higher production volumes, other cost components, particularly labor and raw materials, are expected to become more dominant in the cost structure.

To benchmark the manufacturing cost of NIPS-based GDMs against conventional GDMs, we utilized the cost analysis by James *et al.*,^47^ which models the effect of production scale on conventional GDM costs (Fig. 6d). We extracted a power law scaling relationship (exponent ∼ −0.5) from their data and applied it to our base case of 51 500 m^2^ year^−1^, extrapolating to the same production volume used by James *et al.* (7.6 million m^2^ year^−1^). Our analysis suggests that, at high volumes, NIPS GDMs could be produced at $2.06 per m^2^, which is 55% lower than the $4.60 per m^2^ estimated for conventional GDMs. This significant cost advantage is primarily due to differences in processing: in the James *et al.* model, high-temperature thermal treatments (oxidation, carbonization, graphitization) represent ∼37% of the total cost, whereas our low-temperature carbonization accounts for only ∼5%. Additionally, the NIPS process eliminates the need for paper-making (∼16% of cost) and microporous layer coating (∼12%). Although our projected cost falls below the current raw material cost ($3.83 per m^2^), we expect these material costs to decrease with bulk purchasing at industrial scale.

An alternative way to evaluate the impact of the novel GDM, independent of the techno-economic model, is by translating the observed 16% performance improvement at 0.7 V into a reduction in stack power cost ($ kW_net_^−1^). The performance gained by using the novel GDM allows for a reduced active cell area while maintaining the same stack power output. The smaller active area translates directly into lower material costs for the CL, BP and membrane, while some other elements (*e.g.* cell voltage monitor, MEA assembly, balance of system, and contingency) remain unchanged. As shown in Fig. 6e, this 16% performance enhancement, combined with the lower GDM manufacturing cost, results in an overall 13% reduction in stack power cost.^79^

Overall, our techno-economic model suggests substantial potential for cost reduction in GDM production through the adoption of this alternative manufacturing process. Additionally, the novel microstructure obtained may contribute to lower net stack power costs by enhancing fuel cell performance. However, it would also be important to test these GDM materials at scale under more commercially relevant conditions to assess their durability as it is important to be able to match conventional GDM materials in terms of product life time as well as performance. Furthermore, it is important to emphasize that the current model provides only a rough, order-of-magnitude estimate of actual production costs. To accurately capture both OPEX and CAPEX, more detailed and sophisticated cost modeling – incorporating input from industry stakeholders – is necessary.

## 4. Conclusion

In this work, we present a new strategy for fabricating gas diffusion media (GDM) for low-temperature PEM fuel cells using a scalable, bottom-up approach based on non-solvent induced phase separation (NIPS). By tailoring the polymer solution formulation, casting thickness, and introducing a vapor-induced phase separation step, we created hydrophobic carbon-based GDMs with tunable microstructures and robust mechanical properties. We fabricated and benchmarked two distinct NIPS-derived GDMs - macrovoid-type and sponge-like structures against a commercial state-of-the-art material (Freudenberg H15C14). Despite their stark microstructural differences - and the lack of a dedicated microporous layer - the NIPS GDMs showed comparable or superior electrochemical performance. The macrovoid design outperformed in the kinetic and ohmic regimes, likely due to improved interfacial contact from its smoother surface. However, it exhibited higher mass transport limitations at high current densities, potentially due to elevated hydrophilicity and a dense surface skin layer that restricted gas diffusion. To overcome this, we developed sponge-like GDMs using a vapor induced phase separation pre-step, which yielded thinner, more isotropic structures with open surface porosity. These materials improved mass transport behavior but underperformed in the low-current regime, likely due to increased contact resistance. A third variant – macrovoid GDMs with a laser-ablated top layer – combined the benefits of both designs, significantly improving gas transport and overall fuel cell performance.

A techno-economic analysis confirmed the scalability of the NIPS approach, with an estimated production cost of $24 m^−2^ at pre-industrial scale, and potential for further reduction *via* process optimization and economies of scale. We estimate that implementing this new GDM architecture could reduce total stack power costs by ∼13% at 0.7 V operating voltage, primarily due to improved power density enabling smaller active areas. Overall, this study demonstrates the potential of NIPS-based fabrication to unlock unprecedented microstructural design spaces beyond conventional carbon fiber-based GDMs. By decoupling microstructure from carbon fiber constraints, our approach unlocks new pathways for engineering mass transport properties and interfacial behavior, which are critical to advancing PEMFC performance and cost targets. Future work will focus on optimizing surface properties and internal architecture to further minimize mass transport resistance, improving process scalability and throughput, and leveraging advanced *operando* diagnostics to image multiphase flow behavior. Together, these advances could help accelerate fuel cell adoption in key applications such as heavy-duty transport and stationary power. Beyond applications in low-temperature fuel cells, we envision that this methodology may also be applicable to a broader range of electrochemical technologies involving multiphase flow regimes.

## Author contributions

R. J. H. contributed to the conceptualization, methodology, validation, formal analysis, investigation, data curation, writing – original draft, writing – review and editing, and visualization. R. v. d. L. contributed to methodology, validation, investigation, visualization. R. R. J. contributed to conceptualization, methodology, resources, data curation, formal analysis, writing – review and editing B. L. contributed to methodology, data curation, formal analysis, writing – review and editing. Finally, A. F. C. contributed to the conceptualization, methodology, funding, resources, writing – original draft, writing – review and editing, project administration, and supervision.

## Conflicts of interest

The authors declare no conflict of interest.

## Supplementary Material

EE-018-D5EE03633J-s001

EE-018-D5EE03633J-s002

## Data Availability

The data supporting this article have been included either in the paper or as part of the supplementary information (SI). See DOI: https://doi.org/10.1039/d5ee03633j.
