# Supplementary material for: Designer diffusion media microstructures enhance polymer electrolyte fuel cell performance
Source: Energy Environ Sci. 2025 Oct 14;18(23):10061–77. doi: 10.1039/d5ee03633j (PMC12573437; doi:10.1039/d5ee03633j)
Supplement: EE-018-D5EE03633J-s001 [file EE-018-D5EE03633J-s001.pdf]

Supplementary Information (SI) for Energy & Environmental Science.
This journal is © The Royal Society of Chemistry 2025

Supplementary Information

Designer diffusion media microstructures enhance polymer electrolyte fuel cell performance

Rens J. Horst,[a] Ralph van der Linde,[a] Rémy R. Jacquemond,[a] Baichen Liu[a] and Antoni Forner-Cuenca[a*]

[a]*Electrochemical Materials and Systems, Department of Chemical Engineering and Chemistry, Eindhoven University of Technology, PO Box 513, 5600 MB Eindhoven, Netherlands*

* *Corresponding author: a.forner.cuenca@tue.nl*

S1 Commercial CCM cross-section 2
S2 NIPS and VIPS process overview 2
S3 Fuel cell hardware 3
S4 Skin layer NIPS SEM 4
S5 FH15C14 EDX PTFE distribution 5
S6 NIPS EDX PTFE distribution 5
S7 Additional XPS data 6
S8 Through-plane conductivity 6
S9 XPS analysis of NIPS GDM after laser treatment 7
S10 Residuals of the DRT measurements 7
S11 Capillary flow porometry 8
S12 H_2/N_2 impedance spectra for proton conductivity 9
References 10

S1 Commercial CCM cross-section

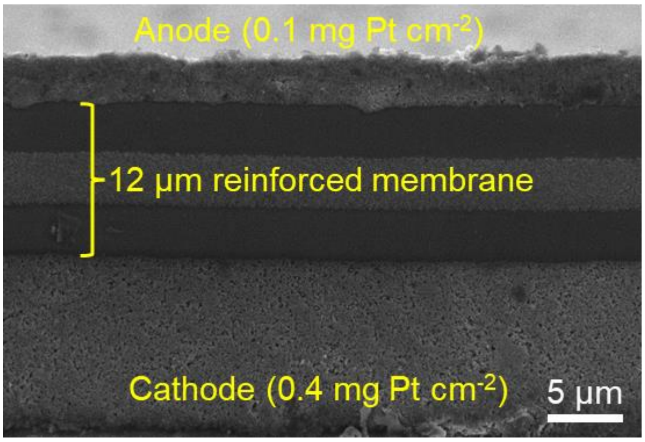

Figure S1: electron micrograph of cryo ion milled cross-section of commercial CCM used for all the measurements in this paper. Top side is the anode (0.1 mg Pt / cm^2), bottom side is the cathode (0.4 mg Pt / cm^2) and the three layers in between are the reinforced proton exchange membrane (M788.12). Cryo ion milling was performed using a Hitachi IM4000II ion miller at -95 °C with 4 kV acceleration voltage, 5s on 3s off, 30° stage swing.

S2 NIPS and VIPS process overview

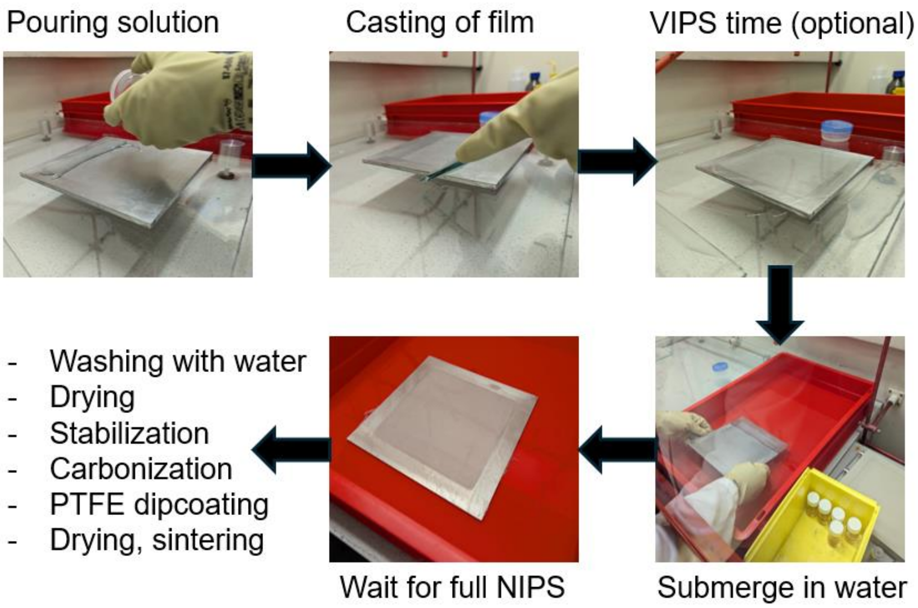

Figure S2: Overview of the polymer casting, VIPS step and NIPS step to give an impression how the PAN membrane is formed in the mold. The mold used here has a depth of 400 µm and an area of 15 x 15 cm^2. During casting it is important not to entrap air in the film, for that reason it is important to move slowly and at an angle as indicated in the picture. For the VIPS step it is important to know the relative humidity or better controlling it, but for our process the lab temperature was fairly constant at around 21 °C and relative humidity during casting was between 60 and 70 % RH. For submersion it important to submerge in one fluent motion as to initiate the NIPS uniformly over the entire film surface. Removal of the NIPS from the mold is achieved by gently removing the excess polymer from the edges of the mold, then the formed membrane will release by itself.

S3 Fuel cell hardware

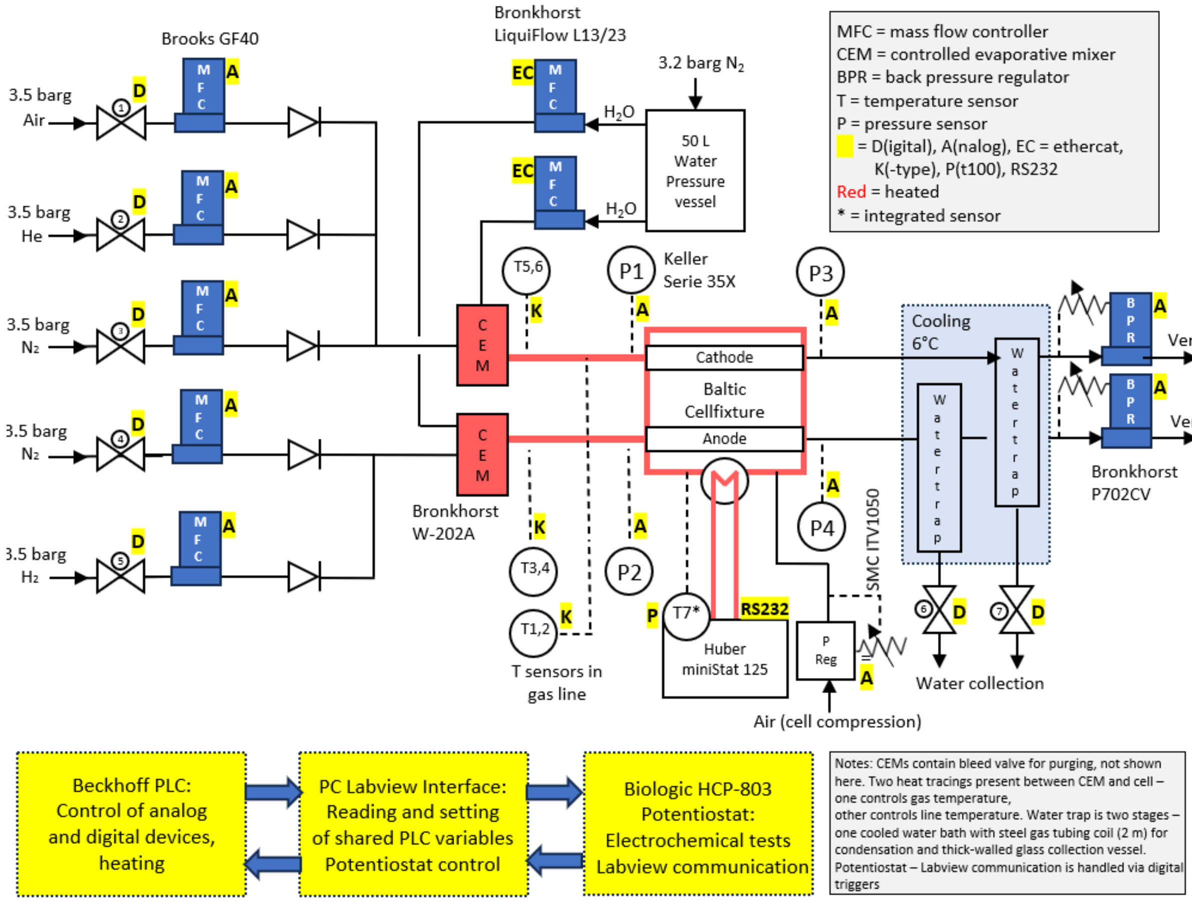

Figure S3: Schematic representation of the single-cell fuel cell test setup used in this paper. The setup was made in-house and consists of a gas conditioning section capable of controlling flow rate and gas composition. A controlled evaporative mixer (Bronkhorst W-202A) per line (anode and cathode) is used to humidify the gasses to a certain % relative humidity. The liquid is pressurized using N_2 gas at 300 mbar below the gas line pressure. This ensures that the gas flow controllers can always inject gas into the CEM mixing valve. The gasses then enter a heated section where that gas temperature is precisely controlled by an in-gas temperature sensor to ensure that no condensation of the injected water vapor takes place. Pressure sensors (Keller Serie 35X) are located before and after the cell to record the cell pressure drop and differential pressure. We employ a cellfixture cf5/100HT and qcF FC25/100 V2.0 support frame (BalticFuelcells GmbH) which is compressed using an automated regulator (SMC ITV1050) controlled by the cell operating pressure. Cell operating pressure is controlled using back pressure regulators (Bronkhorst P702CV) and water transported by the gas coming out of the cell is condensed and collected so that only low dew point gas (6°C) reaches the back pressure controllers. All devices are controlled using a Beckhoff based PLC which is connected to a PC Labview environment as a user interface. The potentiostat controls the Labview environment based on a digital trigger system whenever a new electrochemical technique is performed.

S4 Skin layer NIPS SEM

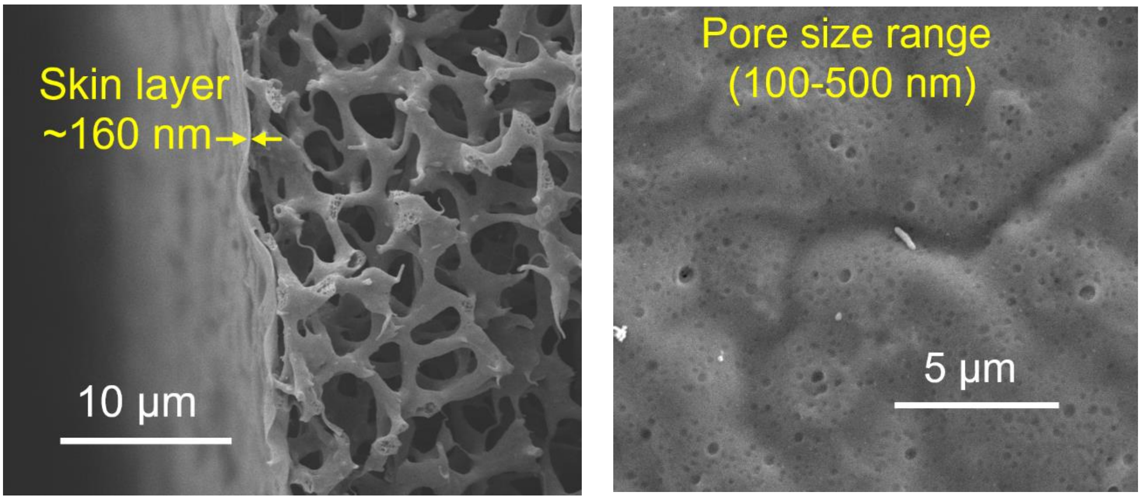

Figure S4: high resolution electron micrograph of a NIPS Macrovoids cross-section looking at the skin layer (left) and a view directly normal to the skin layer (right), showcasing small pores in this skin layer with pore sizes smaller than 1 µm and the minimal thickness of the skin layer as estimated around 160 nm. This image shows that these layers are very thin and not completely dense and should in principle allow for mass transport.

S5 FH15C14 EDX PTFE distribution

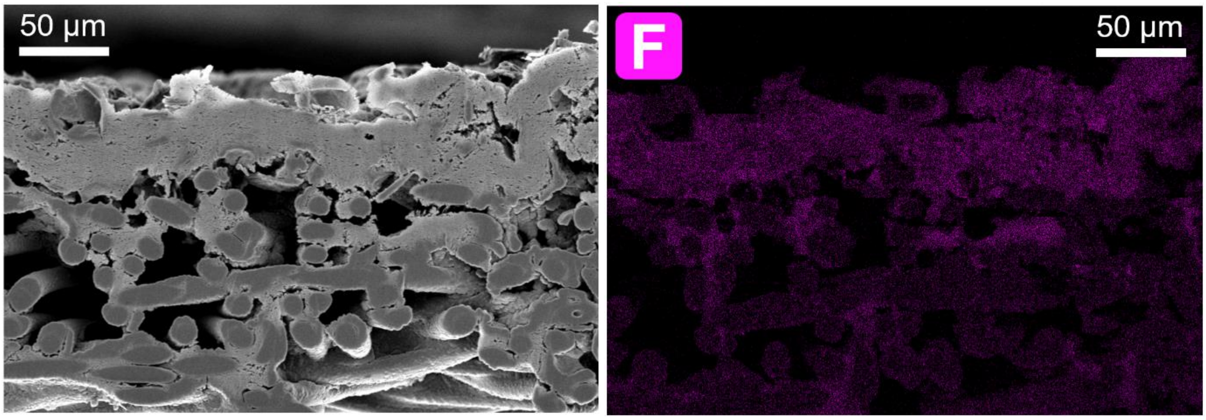

Figure S5: cross-sectional electron micrograph of the baseline GDM (FH15C14) and corresponding EDX fluorine map showing that the PTFE has been distributed throughout the porous GDM structure, but the highest intensity is in the top layer in the image which is the microporous layer (this is where PTFE is used as a hydrophobic binder).

S6 NIPS EDX PTFE distribution

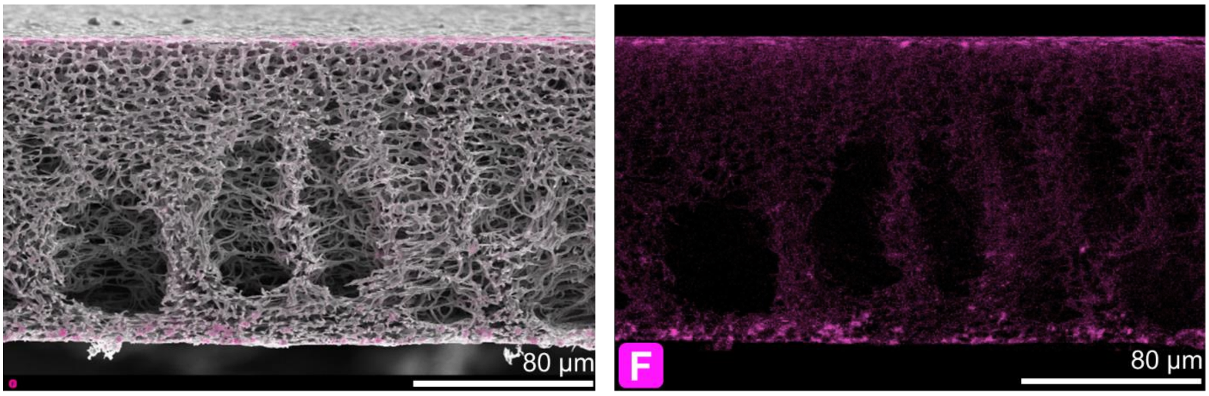

Figure S6: cross-sectional electron micrograph of a hydrophobized macrovoids GDM and corresponding EDX fluorine map showing that the PTFE has been distributed throughout the porous GDM structure, but the top and bottom interface appear to have a higher PTFE content than the internal structure.

S7 Additional XPS data

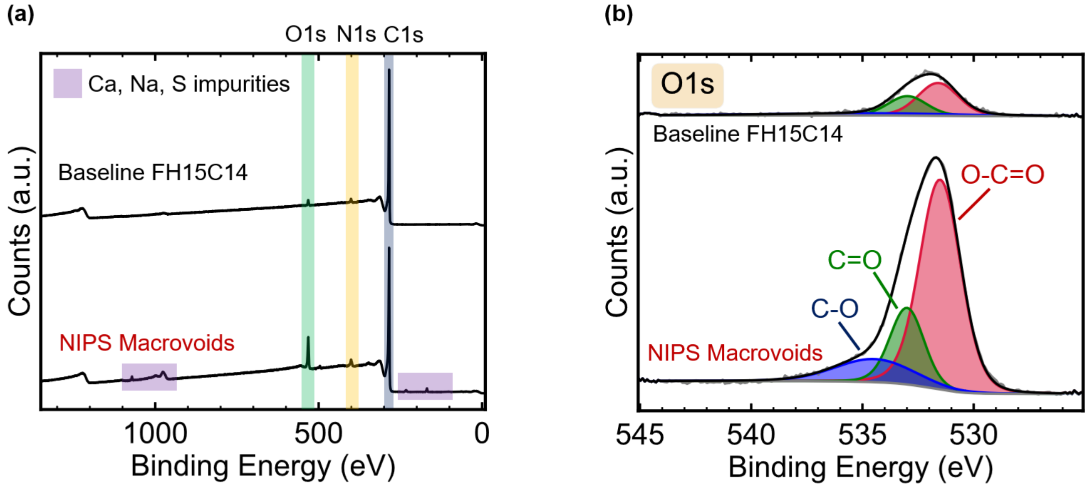

Figure S7: (a) XPS survey spectrum of the FH15 (FH15C14 without MPL or hydrophobization) and the untreated NIPS Macrovoids sample a clear difference in peak intensity for the heteroatoms (O, N) can be observed. Furthermore, the NIPS Macrovoids sample contains some additional impurities, likely because of the washing step with tap water and the carbonization and stabilization ovens that are shared among different research groups/lines. Quantification of the baseline peaks yields C1s 97%, O1s 1.4%, N1s 1.6% vs. respectively 91, 5, and 3 atom-% for the NIPS sample. (b) XPS O1s spectrum showing the differences in oxygen peak intensity and the concomitant deconvolution into different oxygen functionalities. The black line is the resulting fit envelope.

S8 Through-plane conductivity

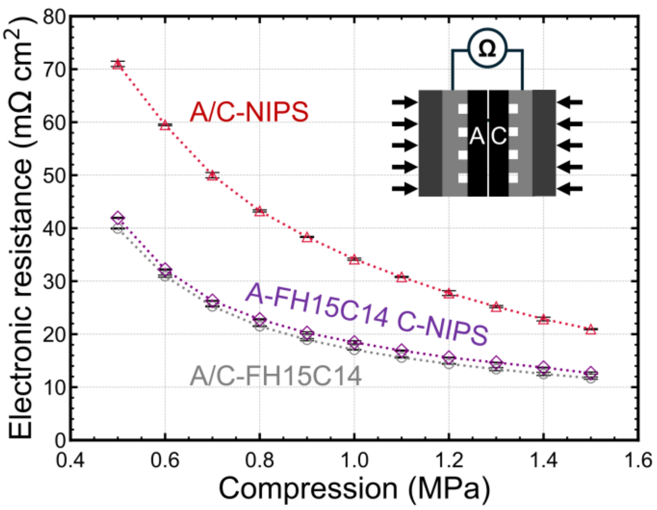

Figure S8: graph of measured electronic resistance of GDM materials under different amounts of compression as measured in our cell used for in-situ testing. The measurements was performed using a sandwich of two GDMs always oriented with the CL-side towards each other. The NIPS samples used were macrovoids NIPS GDMs and A or C stands for the anode and cathode side respectively.

S9 XPS analysis of NIPS GDM after laser treatment

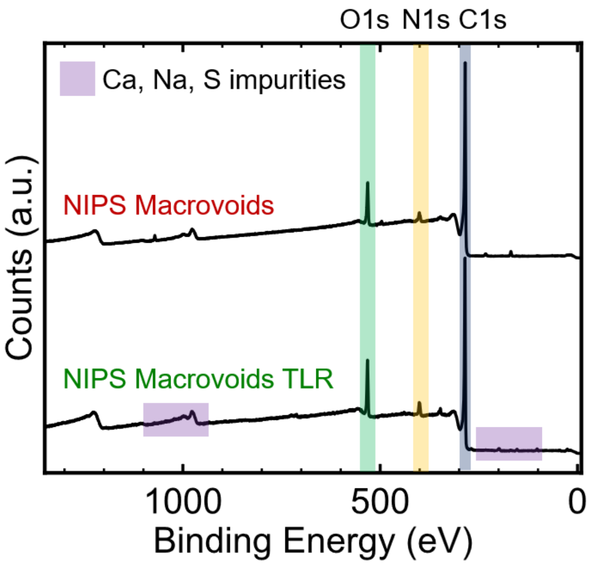

Element (atom %)	C	N	O
Macrovoids	91	3	5
Macrovoids TLR	84	5	11

Figure S9: XPS survey spectra of a post-treated NIPS Macrovoids sample to remove the top layer using a laser (TLR) and an untreated sample to show the difference in heteroatom content caused by the laser treatment.

S10 Residuals of the DRT measurements

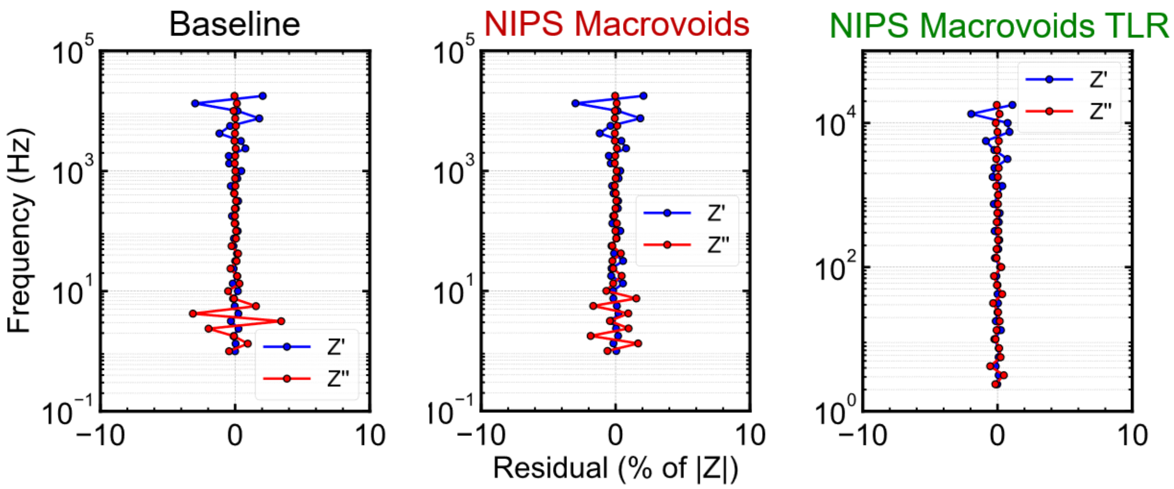

Figure S10: Residuals (% of actual impedance value) as a function of frequency for the data used to obtain the DRT fitting results as presented in the manuscript. Some deviations can be observed in the high frequency (>10,000 Hz) and low frequency region (<1 Hz). High frequency deviations are likely caused by the inductive characteristics of our measurement setup and the low frequency deviations are likely caused by instabilities in the water management of the cell at high current densities (measurement was performed around 0.3V).

S11 Capillary flow porometry

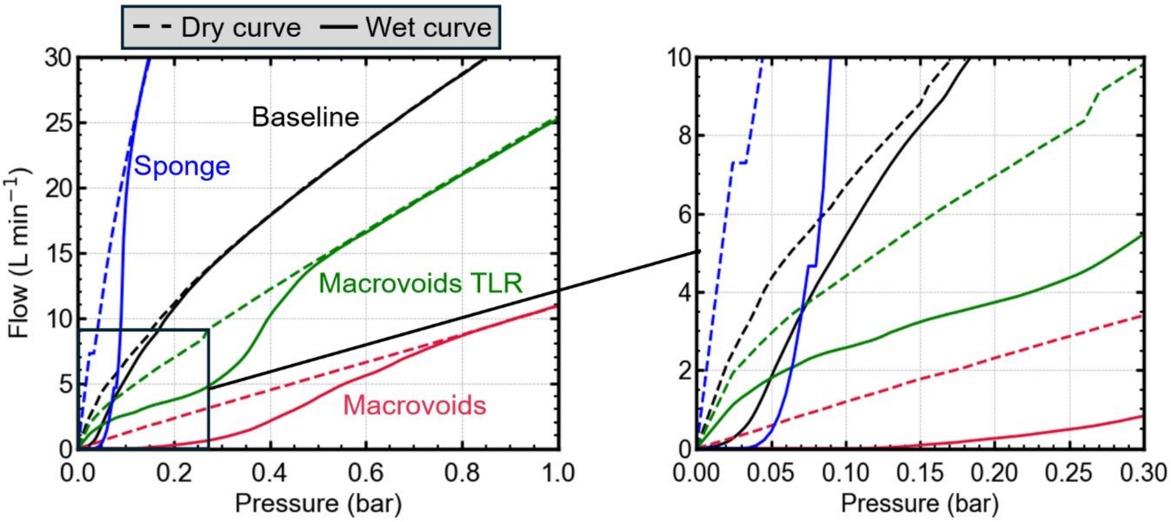

Figure S11: Capillary flow porometry data obtained for all the samples. Capillary flow porometry – porometry was used to measure the GDM permeability and get an indication of the pore size distribution. A Porolux 500 (IB-FT) was used with a 25 mm sample holder and Porofil as wetting liquid, 60 s / bar from 0 to 3 bar.

S12 H_2/N_2 impedance spectra for proton conductivity

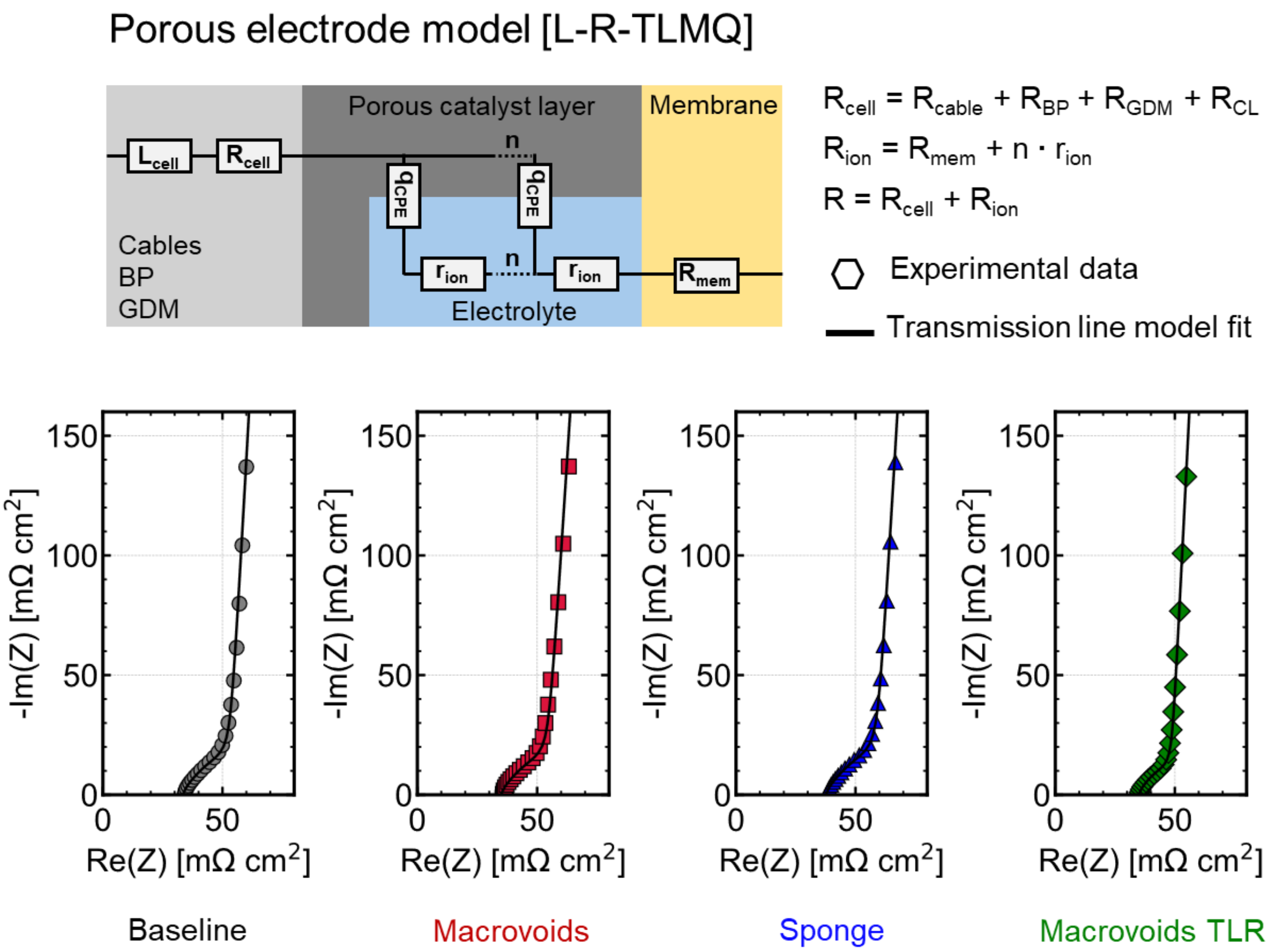

Figure S12 Simplified transmission line model based on that of Landesfeind et al.[1] together with the H_2/N_2 blocking electrode potentiostatic impedance spectroscopy data acquired at a potential of 0.2 V vs. the hydrogen anode with a 10 mV perturbation at frequencies between 100 kHz and 0.1 Hz, 8 points per decade and 3 measurements per point. Data in the graph is a zoom in of the entire data range to clearly show the area of interest for the determination of the proton transport resistance. The data full is fit to a transmission line model for a porous electrode (L-R-TLMQ) to extract the proton transport resistance ($R_{H+} = r_{ion} \cdot n$) as reported in the manuscript. The model assumes that the resistance in the porous electrode consists out of a transmission line of an ionic phase (r_{ion}) and a constant phase element (q_{cpe}). A constant phase element is used to account for the non-ideality in the capacitive behavior (ideal capacitor is a perfectly vertical line).

References

1. Landesfeind, J., Hattendorff, J., Ehrl, A., Wall, W. A. & Gasteiger, H. A. Tortuosity Determination of Battery Electrodes and Separators by Impedance Spectroscopy. *J. Electrochem. Soc.* **163**, A1373 (2016).
